# Supplementary material for: Protein signaling and drug target activation signatures to guide therapy prioritization: Therapeutic resistance and sensitivity in the I-SPY 2 Trial
Source: Cell Rep Med. 2023 Dec 11;4(12):101312. doi: 10.1016/j.xcrm.2023.101312 (PMC10772394; doi:10.1016/j.xcrm.2023.101312)
Supplement: Document S1. Figures S1 and S2 and Table S1 [file mmc1.pdf]

Cell Reports Medicine, Volume *4*

Supplemental information

Protein signaling and drug target activation signatures to guide therapy prioritization: Therapeutic resistance and sensitivity in the I-SPY 2 Trial

Rosa I. Gallagher, Julia Wulfkuhle, Denise M. Wolf, Lamorna Brown-Swigart, Christina Yau, Nicholas O'Grady, Amrita Basu, Ruixiao Lu, Michael J. Campbell, Mark J. Magbanua, Jean-Philippe Coppé, I-SPY 2 Investigators, Smita M. Asare, Laura Sit, Jeffrey B. Matthews, Jane Perlmutter, Nola Hylton, Minetta C. Liu, W. Fraser Symmans, Hope S. Rugo, Claudine Isaacs, Angela M. DeMichele, Douglas Yee, Paula R. Pohlmann, Gillian L. Hirst, Laura J. Esserman, Laura J. van `t Veer, and Emanuel F. Petricoin

Protein signaling and drug target activation signatures to guide therapy prioritization: therapeutic resistance and sensitivity in the I-SPY 2 TRIAL

Rosa I. Gallagher, Julia Wulfkuhle, Denise M. Wolf, Lamorna Brown-Swigart, Christina Yau, Nicholas O'Grady, Amrita Basu, Ruixiao Lu, Michael J. Campbell, Mark J. Magbanua, Jean-Philippe Coppé, I-SPY2 Investigators, Smita M. Asare, Laura Sit, Jeffrey B. Matthews, Jane Perlmutter, Nola Hylton, Minetta C. Liu, W. Fraser Symmans, Hope S. Rugo, Claudine Isaacs, Angela M. DeMichele, Douglas Yee, Paula R. Pohlmann, Gillian L. Hirst, Laura J. Esserman, Laura J. van 't Veer, Emanuel F. Petricoin

SUPPLEMENTAL INFORMATION

Additional resources

More information about the I-SPY 2 platform trial (NCT01042379) and associated resources can be found at https://clinicaltrials.gov/ct2/show/NCT01042379, https://www.ispytrials.org/i-spy-platform/i-spy2 and https://ispypatient.org.

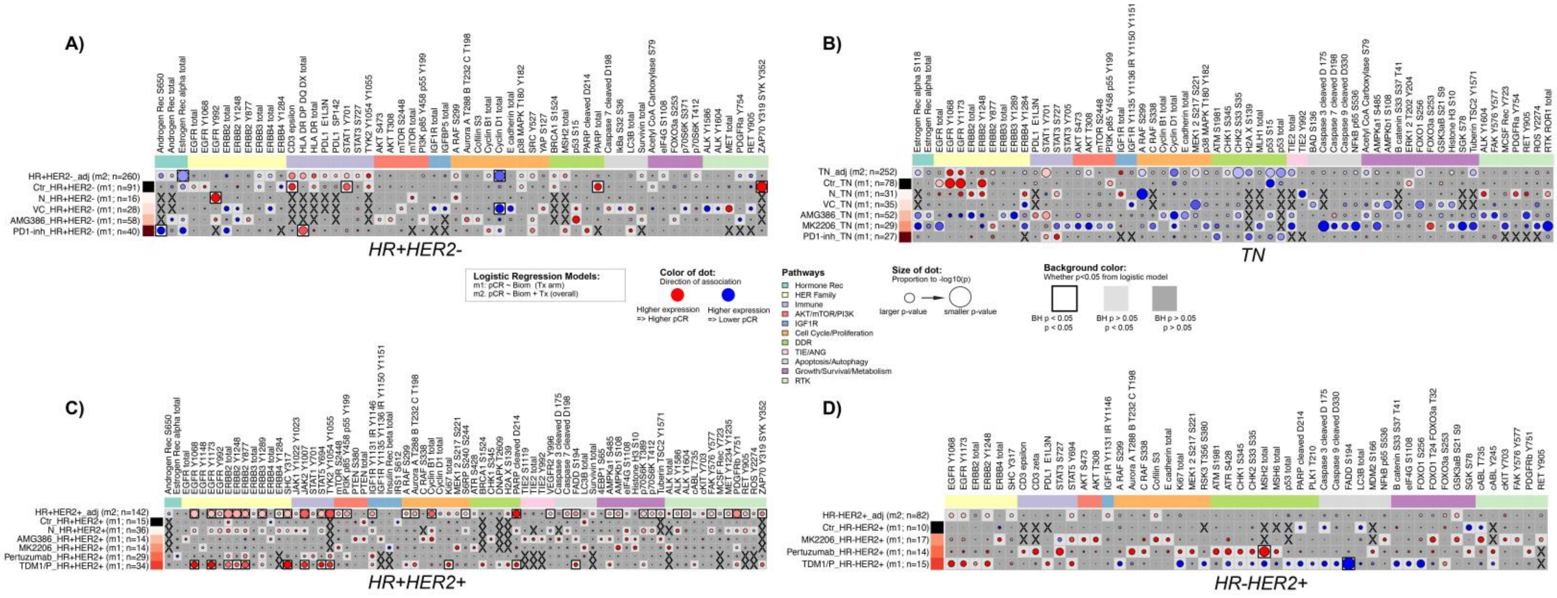

Figure S1. Dot plots of Protein/phosphoprotein expression/activation levels associated with HR/HER2 subtype and pCR. related to Figure 3. Dot plots of A) HR+HER2- subtype, B) Triple negative (TN) subtype, C) HR+HER2+ subtype and D) HR-HER2+ subtype.

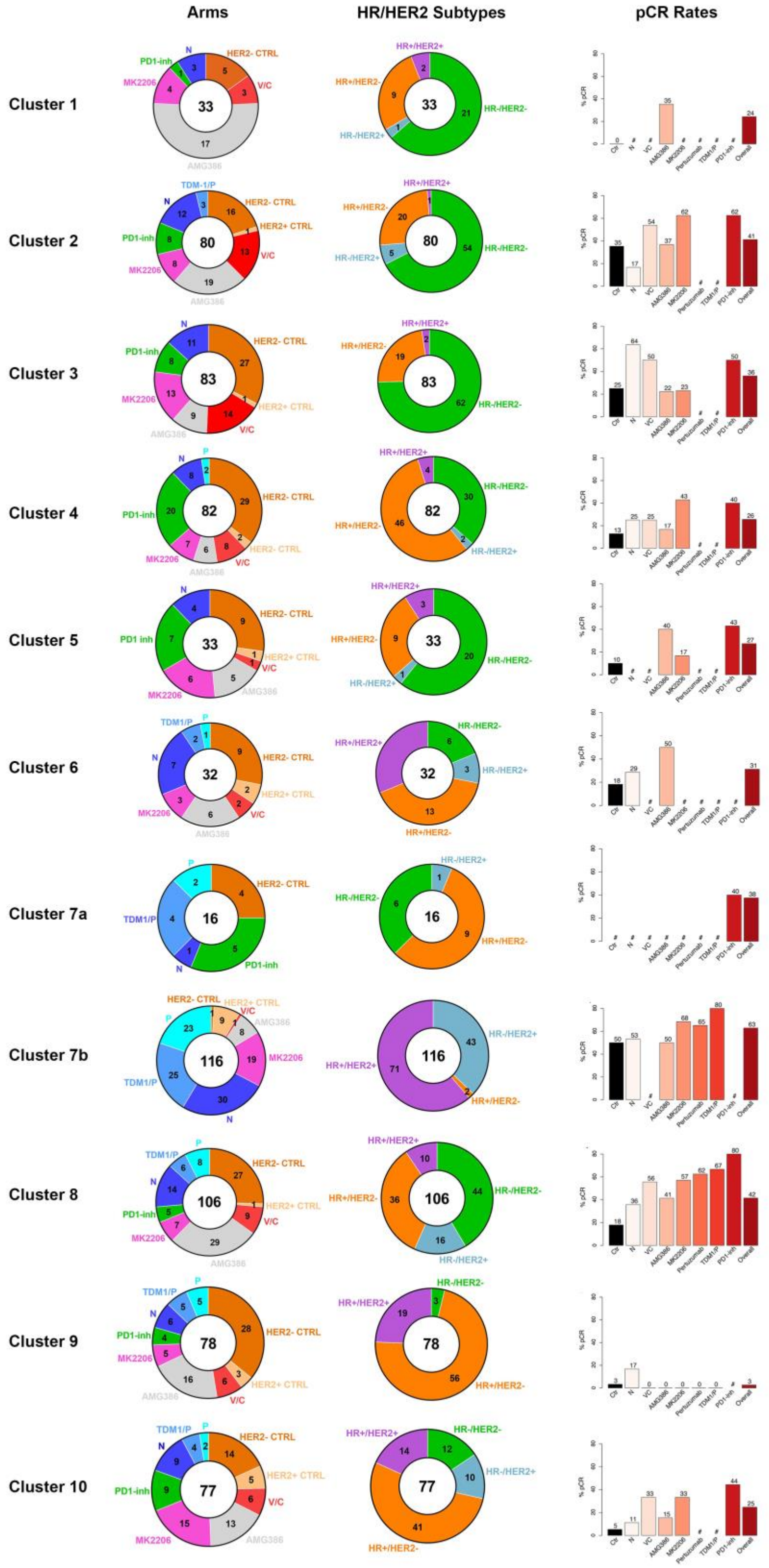

Figure S2. Treatment arm, HR/HER2 subgroup distribution and pCR rates for the 11 protein/phosphoprotein-driven cluster signatures identified, related to Figure 4. Donut plots of treatment arm composition (left), HR/HER2 subtype composition (middle) and pCR rates for each of the therapeutic arms (right) are shown.

	HR+HER2-	TN	HR+HER2+	HR-HER2+	Total
Ctr	91	78	15	10	**194**
N	16	31	36	22	**105**
VC	28	35	0	0	**63**
AMG386	58	52	14	4	**128**
MK2206	27	29	14	17	**87**
Pertuzumab	0	0	29	14	**43**
TDM1/P	0	0	34	15	**49**
PD1-inh	40	27	0	0	**67**
Total	**260**	**252**	**142**	**82**	**736**

Table S1. Patient distribution between arms and HR/HER2 subtypes in the RPPA dataset, related to Figure 1.
