# Supplementary material for: Protein signaling and drug target activation signatures to guide therapy prioritization: Therapeutic resistance and sensitivity in the I-SPY 2 Trial
Source: Cell Rep Med. 2023 Dec 11;4(12):101312. doi: 10.1016/j.xcrm.2023.101312 (PMC10772394; doi:10.1016/j.xcrm.2023.101312)
Supplement: Document S2. Article plus supplemental information [file mmc6.pdf]

Cell Reports
Medicine

Article

Protein signaling and drug target activation signatures to guide therapy prioritization: Therapeutic resistance and sensitivity in the I-SPY 2 Trial

Graphical abstract

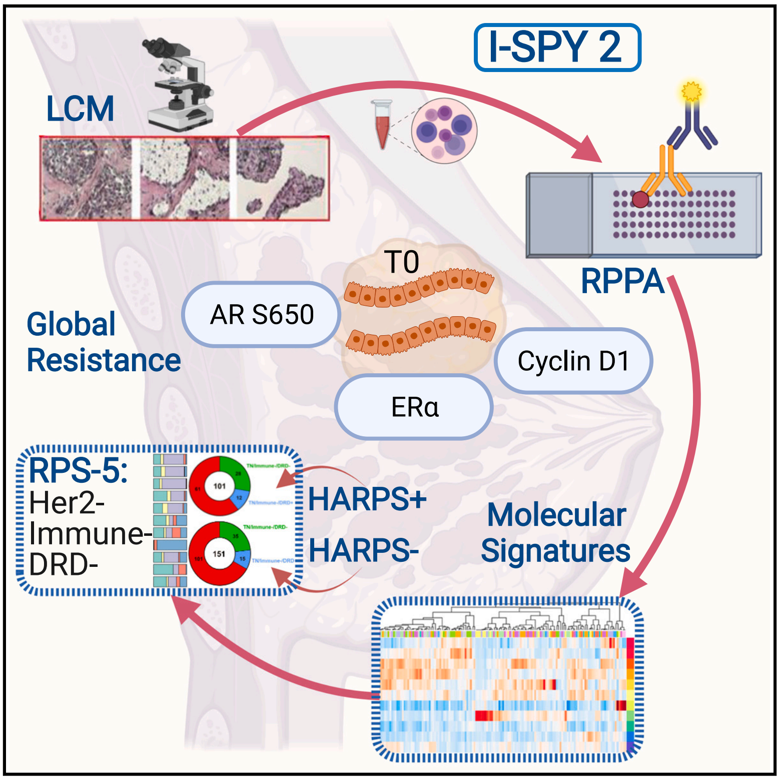

Authors

Rosa I. Gallagher, Julia Wulfkuhle, Denise M. Wolf, ..., Laura J. Esserman, Laura J. van ‘t Veer, Emanuel F. Petricoin

Correspondence

rgallag3@gmu.edu (R.I.G.),
jwulfkuh@gmu.edu (J.W.),
epetrico@gmu.edu (E.F.P.)

In brief

Gallagher et al. identify phosphoprotein and protein-based signatures of global resistance, and treatment-specific and fit-for-purpose response predictive signatures in pre-treatment tumors of I-SPY 2 Trial patients. HER2-EGFR protein activation/phosphorylation signatures could synergize with transcriptomic-based RPS subtyping and provide for better outcomes in patients with triple-negative disease.

Highlights

- LCM-RPPA protein activation mapping identifies signatures of resistance and response
- Cyclin D1, ERα, and AR S650 are biomarkers associating with global resistance
- Identified signatures may be used with RPS to prioritize treatment selection in future arms
- HARPS identifies potential HER2-targeting option to 40% of TNBC patients

Gallagher et al., 2023, Cell Reports Medicine *4*, 101312
December 19, 2023 © 2023 The Author(s).
https://doi.org/10.1016/j.xcrm.2023.101312

CellPress

Cell Reports Medicine

CellPress
OPEN ACCESS

Article

Protein signaling and drug target activation signatures to guide therapy prioritization: Therapeutic resistance and sensitivity in the I-SPY 2 Trial

Rosa I. Gallagher,[1,14,15,*] Julia Wulfkuhle,[1,14,*] Denise M. Wolf,[2,14] Lamorna Brown-Swigart,[2] Christina Yau,[3] Nicholas O'Grady,[3] Amrita Basu,[3] Ruixiao Lu,[4] Michael J. Campbell,[3] Mark J. Magbanua,[2] Jean-Philippe Coppé,[2] I-SPY 2 Investigators, Smita M. Asare,[4] Laura Sit,[3] Jeffrey B. Matthews,[3] Jane Perlmutter,[5] Nola Hylton,[6] Minetta C. Liu,[7] W. Fraser Symmans,[8] Hope S. Rugo,[9] Claudine Isaacs,[10] Angela M. DeMichele,[11] Douglas Yee,[12] Paula R. Pohlmann,[13] Gillian L. Hirst,[3] Laura J. Esserman,[3] Laura J. van 't Veer,[2] and Emanuel F. Petricoin[1,14,*]

[1]Center for Applied Proteomics and Molecular Medicine, George Mason University, Manassas, VA 20110, USA
[2]Department of Laboratory Medicine, University of California, San Francisco, San Francisco, CA 94143, USA
[3]Department of Surgery, University of California, San Francisco, San Francisco, CA 94143, USA
[4]Quantum Leap Healthcare Collaborative, San Francisco, CA 94118, USA
[5]Gemini Group, Ann Arbor, MI 48107, USA
[6]Department of Radiology, University of California, San Francisco, San Francisco, CA 94143, USA
[7]Department of Surgery, Mayo Clinic, Rochester, MN 55905, USA
[8]Department of Pathology, University of Texas MD Anderson Cancer Center, Houston, TX 77030, USA
[9]Division of Hematology/Oncology, University of California, San Francisco, San Francisco, CA 94158, USA
[10]Lombardi Comprehensive Cancer Center, Georgetown University, Washington, DC 20007, USA
[11]Perelman School of Medicine, University of Pennsylvania, Philadelphia, PA 19104, USA
[12]Department of Medicine, University of Minnesota, Minneapolis, MN 55455, USA
[13]Department of Breast Medical Oncology, University of Texas MD Anderson Cancer Center, Houston, TX 77030, USA
[14]These authors contributed equally
[15]Lead contact
*Correspondence: rgallag3@gmu.edu (R.I.G.), jwulfkuh@gmu.edu (J.W.), epetrico@gmu.edu (E.F.P.)
https://doi.org/10.1016/j.xcrm.2023.101312

SUMMARY

Molecular subtyping of breast cancer is based mostly on HR/HER2 and gene expression-based immune, DNA repair deficiency, and luminal signatures. We extend this description via functional protein pathway activation mapping using pre-treatment, quantitative expression data from 139 proteins/phosphoproteins from 736 patients across 8 treatment arms of the I-SPY 2 Trial (ClinicalTrials.gov: NCT01042379). We identify predictive fit-for-purpose, mechanism-of-action-based signatures and individual predictive protein biomarker candidates by evaluating associations with pathologic complete response. Elevated levels of cyclin D1, estrogen receptor alpha, and androgen receptor S650 associate with non-response and are biomarkers for global resistance. We uncover protein/phosphoprotein-based signatures that can be utilized both for molecularly rationalized therapeutic selection and for response prediction. We introduce a dichotomous HER2 activation response predictive signature for stratifying triple-negative breast cancer patients to either HER2 or immune checkpoint therapy response as a model for how protein activation signatures provide a different lens to view the molecular landscape of breast cancer and synergize with transcriptomic-defined signatures.

INTRODUCTION

Breast cancer is the second leading cause of cancer deaths in women in the US.[1] with nearly 298,000 new cases projected to occur in 2023.[2] Most breast cancer cases exhibit heterogeneous populations of tumor cells generating different clinical behaviors and complex biologies that limit therapeutic strategies.[3,4] Neoadjuvant chemotherapy trials, such as I-SPY 2, facilitate the assessment of sensitivity to different breast cancer therapeutic agents by measuring patients' pathologic complete response (pCR), which provides valuable prognostic information and can inform the need for additional adjuvant therapy.[5]

The I-SPY 2 Trial is a multicenter, phase II, adaptive neoadjuvant therapy trial, which, in addition to rapidly identifying new therapies that could provide benefit in the neoadjuvant setting, has the aim to utilize a multi-omic biomarker approach to identify molecular signatures of response and resistance beyond HR/HER2 status (Figure 1A). Such efforts could potentially uncover new therapeutic strategies for overcoming *de novo* resistance and identify subpopulations of patients optimally tuned to best response in a modern treatment landscape.[6] Recently, we have described mRNA-based response predictive subtypes (RPSs) based on gene expression signatures and, if used to allocate treatment decisions, are

This is an open access article under the CC BY license (http://creativecommons.org/licenses/by/4.0/).

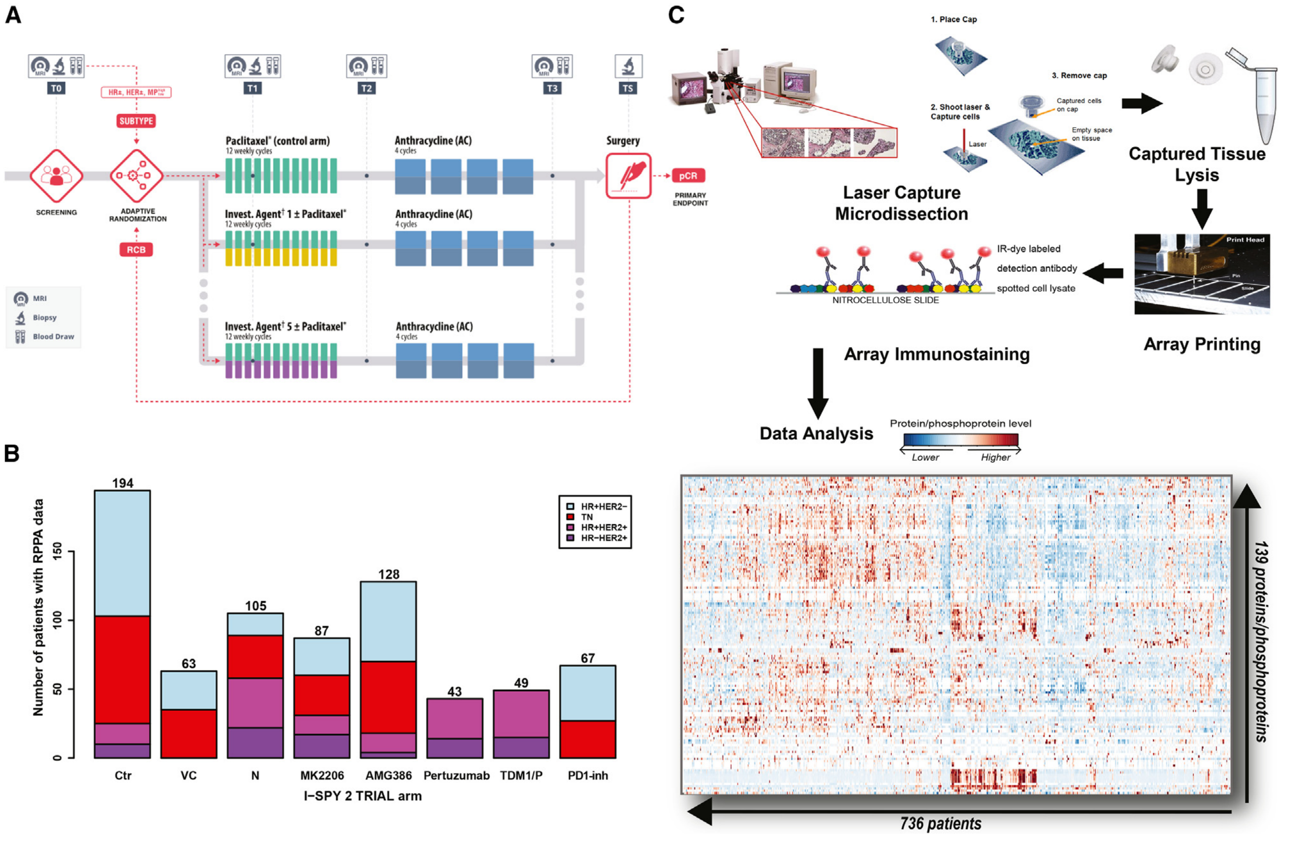

Figure 1. I-SPY 2 Trial design, RPPA workflow, and patient distribution

(A) I-SPY 2 Trial schematic.[6]

(B) Patient number distribution by trial arm and HR/HER2 status in the reverse phase protein array (RPPA) dataset. Ctr, control; N, neratinib; PD1-inh, PD1 inhibitor; TDM1/P, TDM1 + pertuzumab; VC, veliparib + carboplatin.

(C) RPPA workflow (image modified from Loebke et al.[9]).

predicted to improve patient response and outcome.[6] However, even with RPS-based categorization there are subsets of patients with extremely low pCR rates for all tested agents to date. Patients who do not achieve pCR in the neoadjuvant setting have the poorest recurrence-free survival compared with women who achieve pCR.[5,7,8]

While exploration of the genomic/transcriptomic landscape is of obvious importance in discovering new targets, the biochemical mechanism of action (MOA) of nearly all precision therapeutics is proteomic based, involving modulation of protein expression and/or function, or binding to protein receptors and delivering therapeutic payloads. In previous I-SPY and other targeted therapy studies, reverse-phase protein array (RPPA) analysis successfully identified specific protein/phosphoprotein markers that predicted response to targeted therapies missed by current genomic or transcriptomic biomarkers.[10–14] Here, we continue and expand this protein/phosphoprotein analytic work, which complements the mostly transcriptional-based analysis in our companion subtyping paper.[6]

Recent investigations of tissue-based proteomic biomarkers associating with breast cancer neoadjuvant therapy clinical response have relied solely on either mass spectrometry-based analysis of whole tissue lysates[15,16] or on the evaluation of individual biomarkers such as PDL1,[17] CAIX,[18] or Ki67.[19] Our investigation and analysis represent a large clinical study set of proteomics data for treatment-naive tumors from breast cancer (n = 736) that utilizes laser capture microdissection (LCM)-enriched tumor epithelium for analysis. These data are annotated with HR/HER2 and RPS subtyping, treatment history, outcome (pCR), and distant recurrence-free survival (DRFS) information, and we use this large relational dataset to gain insight into mechanisms of treatment specific and global resistance, uncover "fit-for-purpose" predictive (treatment specific/MOA specific) protein expression and signaling activation signatures, and identify druggable protein/phosphoprotein targets and pathways that correlate with lack of pCR and associate with overall prognosis in tumors from patients who do not achieve pCR. This protein expression and signaling activation-based paper and its predominantly gene expression/subtyping companion publication[6] are complemented by the public release of the I-SPY2-990 mRNA/RPPA data resource that includes gene expression data for 990 breast cancer patients and protein/phosphoprotein data for 736 patients with treatment and response data in up to 10 arms of the trial.

CellPress
OPEN ACCESS

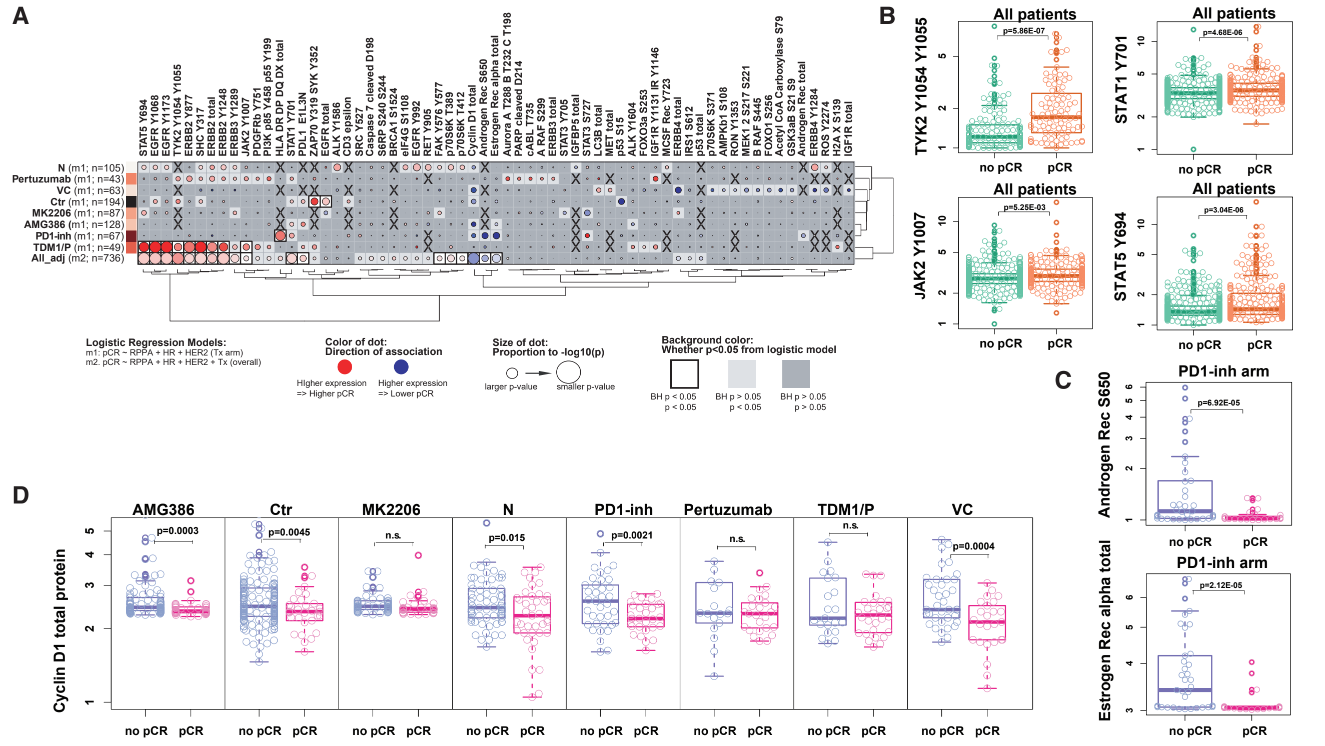

Figure 2. Association of protein/phosphoprotein expression with pCR by arm

(A) Dot plot of protein/phosphoprotein analytes (columns) having significant associations with pCR in one or more treatment arm(s) of the I-SPY 2 Trial or across 8 arms (rows); X, data not available.

(B) Boxplots of TYK2 Y1054/Y1055 and STAT1 Y701 (top) with JAK2 Y1007 and STAT5 Y694 (bottom) expression by pCR status across all arms. Green, no pCR; orange, pCR.

(C) Boxplots of AR S650 (top) and ER total (bottom) by pCR status in the PD1-inh arm. Blue, no-pCR; pink, pCR.

(D) Boxplot of cyclin D1 expression within each arm by pCR status. Blue, non-pCR; pink, pCR. Unadjusted p values annotated within each graph; n.s., not significant; Boxes show median and 25th to 75th interquartile range (IQR). Whiskers denote largest/smallest values within 1.5× the IQR.

RESULTS

Predictive protein/phosphoprotein biomarkers of global sensitivity/resistance in 8 treatment arms across the I-SPY 2 Trial

A total of 736 patients from 8 treatment arms of the I-SPY 2 Trial (control [Ctr], 194; neratinib [N], 105; veliparib/carboplatin [VC], 63; AMG386, 128; MK2206, 87; trastuzumab/pertuzumab [P], 43; TDM1/P, 49; and a PD1 inhibitor [PD1-inh], 67) were included in this analysis (Figure 1B). Thirty-five percent (260/736) of tumors were HR+ HER2−, 34% (252/736) triple negative (TN), and 30% (224/736) HER2+ (11% HR− and 19% HR+) (Table S1). The RPPA component of the I-SPY2-990 mRNA/RPPA data resource contains protein/phosphoprotein data combined across three arrays from the pre-treatment tumor epithelia from these patients. A total of 139 proteins and phosphoproteins, representing key cancer signaling pathways including DNA repair deficiency (DRD), cell cycle/proliferation, PI3K/AKT/mTOR signaling, receptor tyrosine kinases (RTKs), immune and survival signaling were quantitatively measured by RPPA-based protein expression and signaling activation analysis (Figure 1C). Clinical data included HR, HER2, and MP status, response (pCR or no pCR), and treatment arm (Table S2). These data are publicly available in NCBI's Gene Expression Omnibus (SubSeries GSE196093 [RPPA] from SuperSeries GSE196096, which also contains gene expression data) and through the I-SPY 2 Google Cloud repository (http://www.ispytrials.org/results/data).[6]

We evaluated the association of protein/phosphoprotein expression with pCR in enriched tumor epithelium for each of the experimental treatment and shared control arms across all assessable patients. Association analysis across all treatment arms and patients revealed 18 biomarkers significantly associated with response (Figure 2A). All 8 trial arms had RPPA proteins/phosphoproteins that were at least nominally associated with pCR. Experimental arms targeting HER2 had the most associations (e.g., 19 in N, 18 in TDM1/P and 16 in P), whereas the AMG386, MK2206, and PD1-inh arms had the fewest (5–7), despite inclusion of HER2+ patients in two of these arms (Figure 2A; Table S3).

We found that increased expression/activation of three protein/phosphoprotein biomarkers associated with non-pCR and, thus, global resistance in the overall trial population: cyclin D1, estrogen receptor alpha (ERα), and androgen receptor S650 (AR S650) (Figure 2A). For total ERα and AR S650, the association with non-pCR was also seen in the PD1-inh arm, along

with total AR (Figures 2A and 2C). Elevated expression of cyclin D1, a cell-cycle protein implicated in ER-mediated DNA damage repair, cell-cycle arrest, and survival via repression of apoptosis, also nominally associated with non-pCR in the VC, Ctr, AMG386, PD1-inh, and N arms in a model adjusting for HR/HER2 status and Tx ($p < 0.05$) (Figure 2D).

Increased expression of HER2 family proteins/phosphoproteins associated with pCR in the population as a whole. Not surprisingly, HER2 pathway signaling signatures were nominally positively associated with pCR in N, P, and TDM1/P individually (Figure 2A, rows 1, 2, and 8; Table S3) as described previously[10,14]; however, only TDM1/P associations remained significant following p value correction (Figure 2A, row 9; Table S3). Consistent with our previous findings, co-activation of HER2 and EGFR, measured by ERBB2 Y1248 and EGFR Y1173, associated with response to N[14] combined with elevated expression of additional p-RTKs/sites (EGFR Y992, ERBB4 Y1284, ALK Y1586, and RET Y905) associating with pCR in the same treatment arm (Figure 2A, row 1; Table S3).

We also observed immune-related activation signatures associating with pCR in this analysis. Phosphorylated immune-related proteins STAT1 Y701, STAT5 Y694, as well as activation of the upstream JAK2 and TYK2 kinases that regulate STAT phosphorylation associated with response in the population as a whole, along with PDL1 (Figures 2A and 2B). The STAT family proteins are involved with most anti-tumor immune responses mainly through the JAK-STAT signaling pathway,[20] confirming our observations that immune biomarker expression is higher in patients achieving pCR. In the PD1-inh arm, HLA-DR/DP/DQ/DX expression was found significantly elevated in patients achieving pCR (Figure 2A, row 7; Table S3); this observation was reported previously, in addition to STAT1 Y701 expression positively associating with response to PD1-inh (Figure 2A).[11,21] In the MK2206 treatment arm, expression of STAT3 S727 was negatively associated with pCR, while STAT5 Y694 expression showed positive association with pCR in the same arm ($p < 0.05$) (Figure 2A, row 5; Table S3).

Druggable targets associated with HR/HER2 subtypes

Given the known biological differences between receptor subtypes in breast cancer, and because therapeutic arm assignment in I-SPY 2 is driven by tumor HER2 status, we investigated differences in protein/phosphoprotein activation profiles by HR/HER2 and explored associations with pCR within each subtype across all arms. Our results showed that HER2+ subtypes (HR–HER2+ and HR+HER2+) had much higher relative expression levels of ERBB2, pERBB2, pEGFR, and other HER family proteins/phosphoproteins than in HER2– subtypes as expected, and HR–HER2+ tumors had the highest pCR rate (62%, n = 82) of all patient subsets (Figure 3A). HR+ subtypes (HR+HER2– and HR+HER2+) were characterized by high relative expression levels of endocrine receptor proteins (ERα/AR S650), ERBB3/4, PTEN, and IGF1R with HR+HER2– tumors having the lowest pCR rate (18%, n = 260) (Figure 3A). In addition to the expected low levels of endocrine and HER– family signals, TN cancers were characterized by high p53, proliferation, DNA repair deficiency, and immune-related analytes, with a pCR rate of 40% (n = 252). Although TN and HER2+ cancers are "immune-hot,"[22] we observed unexpected variances in immune signaling proteins/phosphoproteins between these two receptor subtypes. For example, STAT5 Y694 and TYK2 Y1054/Y1055 were higher in HER2+ cancers (Figure 3A, red arrows), whereas PD1/PDL1 expression levels were high in TN cancers (Figure 3A, blue arrows). Activation of STAT3 S727 expression was relatively high in both subtypes (Figure 3A, green arrow).

We explored associations with pCR and found that activation of HER2 signaling (ERBB2 Y1248, SHC Y317, EGFR Y1068, and EGFR Y1173) significantly or nominally associated with response in HER2+ tumors as anticipated (Figure 3B, columns 1, 2, and 4; Table S3). Within the HR+HER2– patient subset, high levels of ERα, AR S650, and cyclin D1 significantly associated with non-response (Figures 3B, column 6, and 3C; Table S3). In the HR+HER2– subtype, we observed that RPPA-based ER measurements and JAK-STAT signaling proteins have the same effect across arms (e.g., high ER correlated with resistance; and high JAK-STAT activation correlated with sensitivity) (Figure S1A). We found that HR–/HER2+ tumors from patients treated with TDM-1/P who did not achieve pCR had activation of PLK1 T210 along with significant activation of its direct kinase substrate FADD S194 as well as increased activation/expression of other PLK1 pathway-linked DRD signaling proteins: total MSH2, ATR S248, CHK1 S345, and CHK2 S33/S35 (Figure S1D).

No proteins/phosphoproteins measured by RPPA were significant in the TN subset as a whole after p value correction. As we reported previously,[14] co-activation of EGFR Y1173 and ERBB2 Y1248 in TN tumors associated with response to N. Elevated levels of immune analytes (STAT1 Y701) associated with response to PD1-inh and AMG386, and in MK2206 we found numerous immune markers with high expression levels associated with non-pCR (Figure S1B).[13] Within the HR+HER2+ patient subset, TDM1/P treatment had elevated levels of proteins/phosphoproteins significantly associated with pCR (Figure S1C) and, by contrast, in the small HR–HER2+ subset, nearly all the remaining significant protein biomarkers in the TDM1/P arm associated with resistance other than the activated HER family and immune marker JAK-STAT signaling (Figure S1D).

Druggable targets revealed by protein pathway activation signatures

Because our data were generated from treatment-naive samples, we were curious whether clustering of the baseline protein signaling architecture could reveal associations of signaling activation signatures and potential druggable targets within individual groups agnostic of HR/HER2 status or other stratifying characteristics. Unsupervised hierarchical clustering analysis defined 11 unique signaling-based clusters, many with activation profiles pointing to potential druggable targets representing drug classes employed in the I-SPY 2 Trial to date (Figure 4).

In keeping with the fact that treatment assignment was based on HER2 status and not underpinning signaling characteristics, these protein/phosphoprotein-driven signatures were heterogeneous in treatment modality (Figure 4). A number of clusters were enriched for other biomarker classifiers assessed in the trial,

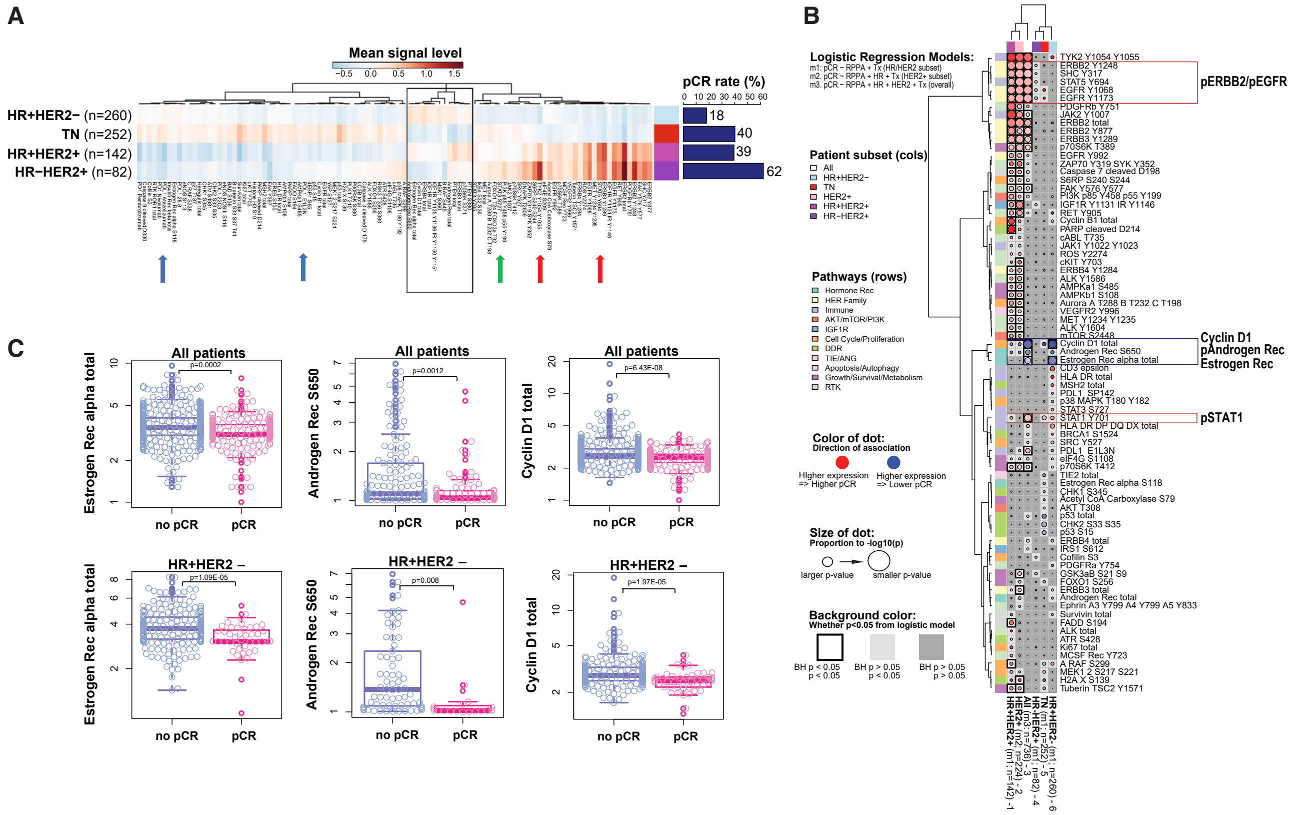

Figure 3. Protein signaling pathway activation-based characterization of receptor subtypes

(A) One-way clustering of analytes with a significant expression difference in at least one subtype pair. Mean intensity values for each endpoint within each subtype were calculated and used for clustering. Mean values from blue to red represent low to high. Bar graph: pCR rate (%) for each subtype across all arms in the study. Blue arrows, PD1/PDL1; green arrow, STAT3 S727; and red arrows, STAT5 Y694 and TYK2 Y1054/Y1055 expression levels.

(B) Association dot plot of protein/phosphoprotein analytes (rows) having significant association with pCR in one or more HR/HER2 subtypes and/or across all subtypes.

(C) Boxplots for total ERα (left), AR S650 (center), and cyclin D1 total (right) in all patients (upper) and the HR+HER2− subset (lower) demonstrating associations with non-pCR. Blue, no pCR; pink, pCR. Boxes show median and 25th to 75th IQR. Whiskers denote largest/smallest values within 1.5× the IQR.

such as HER2/ER receptor subtype/biology, MammaPrint status (MP), PAM50, BluePrint (BP) expression subtype, and the RPS-5 signature (Figure 4; Table S2). For example, (signaling clusters) 1–3 were dominated by patients that were HER2-, MP2, PAM50-basal, and BP-basal subtype. Cluster 7b was mainly comprised of tumors that were in the HER2+ subset and RPS-5 HER2+/BP-Her2_or_Basal (Figures 4, 5A, and S2). Tumors in cluster 9 consisted of mostly HR+HER2− and some HR+HER2+ patients. These tumors were a mix of LumA and LumB by PAM50, and HER2−/Immune−/DRD− by RPS-5, with some HER2+/Luminal and HER2−/Immune+ tumors represented (Figures 4 and 5A).

Relationships between the signaling-based clusters and pCR revealed striking differences in response rates. The protein pathway signature with the highest pCR rate was cluster 7b (63%, n = 116) and was dominated by patients with HER2+ tumors and many who received HER2-directed therapy (N, TDM1/P, and P) (Figures 5A and S2). Cluster 9 was comprised of patient tumors from every treatment arm and had the lowest pCR rate (3%, n = 78) of all the signaling-defined clusters. More than half the patients in this cluster received backbone chemotherapy (Ctrl) or the TIE2 inhibitor AMG386 (44/78) (Figures 5A, 5B, and S2).

We next examined the underlying signaling architecture driving cluster formation by distilling individual analyte data within each cluster to a population mean to better visualize any cluster-specific differences in the signaling patterns (Figure 5A). While some clusters exhibited heterogeneous signaling landscapes, a number of them demonstrated clear patterns of signaling activation with molecularly driven relationships to drug targets, such as HER2 signaling. Cluster 7 was divided into two sub-clusters (7a and 7b) with cluster 7a (n = 16) showing elevated levels of pTIE2 and activation of growth/survival/metabolism pathways (Figure 5A). Cluster 7b (n = 116) was characterized by tumors with elevated levels of HER family signaling activation and a high pCR rate (63%), reflecting the success of the HER2-targeted agents (N, P, TDM1/P) given to patients in this predominantly HR+HER2+ (and HER2 activated) cluster (Figures 4, 5A, and S2, cluster 7b). However, since protein signaling expression was not used as a biomarker strategy for treatment assignment, the underlying tumor signaling signatures in each cluster did not strictly align with the MOAs of treatments

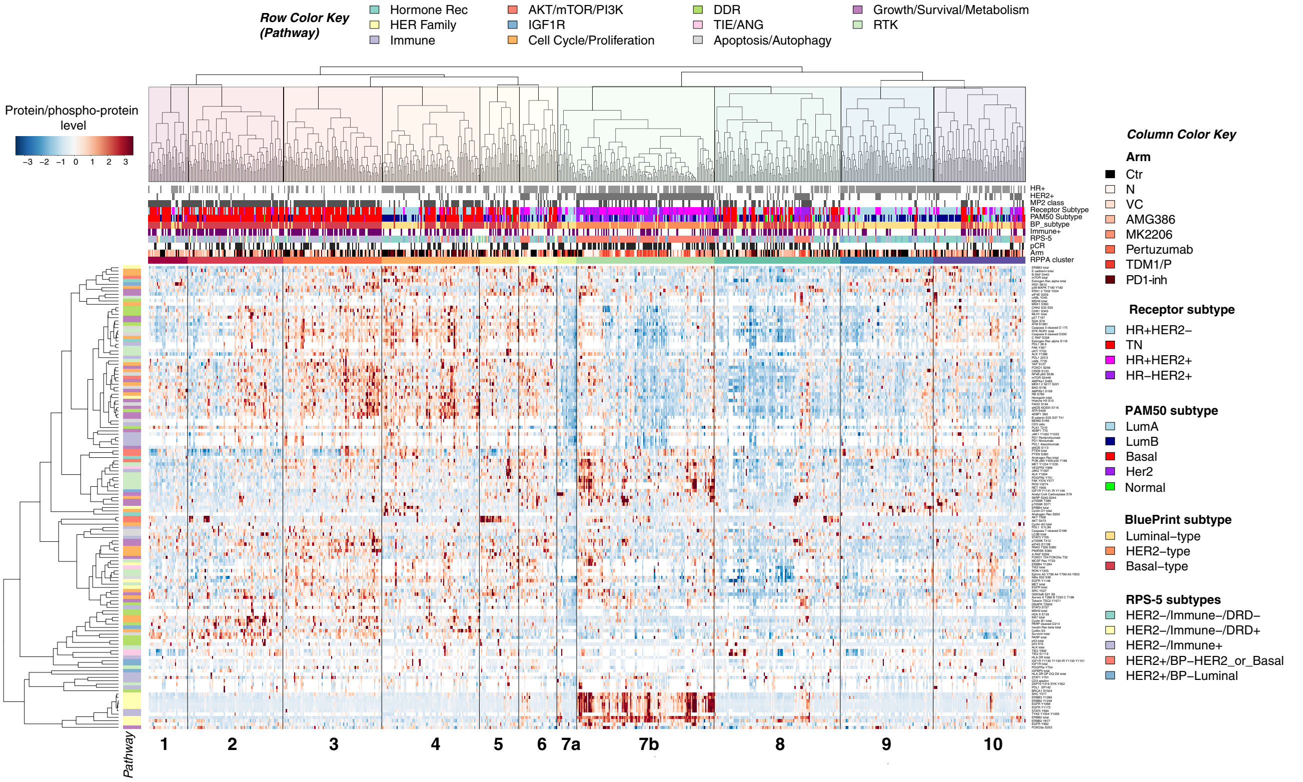

Figure 4. Druggable targets revealed by protein pathway activation clusters

Two-way, unsupervised hierarchical clustering map of protein/phosphoprotein analytes (rows) and 736 patients (columns) comprising the RPPA dataset demonstrating 11 distinct signaling-based clusters. Heatmap color scale: red/white/blue, higher/intermediate/lower levels of expression.

represented. For instance, cluster 7a was characterized by tumors with elevated levels of TIE2 activation, which was associated with AMG386 response in I-SPY 2,[23] yet no patients in this cluster were randomized to the AMG386 treatment arm. Several key druggable targets defined the signaling architecture of a number of the cluster signatures. For example, we observed distinct high expression of immune/cytokine signaling-related proteins HLA-DR, PDL1, and STAT3 Y705 in cluster 1 that indicate potential response to immune checkpoint inhibitors,[11,21] while increased activation/phosphorylation of AKT (S473 and T308) was a main component of the signaling architecture in cluster 5, suggesting that AKT inhibitors could be a therapeutic option for these patients.[12,13]

Identification of pathway signatures and druggable targets associated with poor response

Because cluster 9 had the lowest pCR rate of all clusters (Figures 4, 5A, and S2), we thought it important to investigate the signaling characteristics of this cluster to identify a rationale for selecting targeted agents beyond those tested in the I-SPY 2 Trial thus far. Cluster 9 was comprised of tumors classified as LumA and LumB by PAM50, BP-Luminal, RPS-5 HER2−/Immune−/DRD− and was characterized by elevated levels of cyclin D1, ERα, and AR S650, the global resistance biomarkers identified in this study (Figures 4 and 5A). Given that this cluster was largely composed of HR+HER2− (56/78) and HR+HER2+ (19/78) tumors, we also analyzed these subtypes for potential signaling differences among all other clusters. Of the 139 protein/phosphoproteins measured in our study, 65/139 (47%) and 23/139 (16%) analytes differed significantly (BH LR $p < 0.05$) in cluster 9 from the signaling profiles in all other clusters within HR+HER2− and HR+HER2+ subtypes, respectively (data not shown). Both subtype groups in cluster 9 were characterized by high cyclin D1 and low immune/cytokine-related signaling. However, there were also some differences, as the HR+HER2− subset was characterized by high ERα, and the HR+HER2+ subset by elevated levels of AR S650 (data not shown).

Identification of pathway signatures and druggable targets associated with poor prognosis in non-responding patients

Because we had long-term DRFS follow-up data for many of the patients within our study set (96%; 709/736), we sought to understand the signaling characteristics of tumors from non-responding patients in each cluster, including those with the lowest overall pCR rates such as cluster 9, where patients did not achieve pCR from any I-SPY 2 agent classes included in this analysis. Specifically, we wondered whether non-response for patients in cluster 9 predicted poor long-term outcome, or whether these patients simply have relatively quiescent cancers

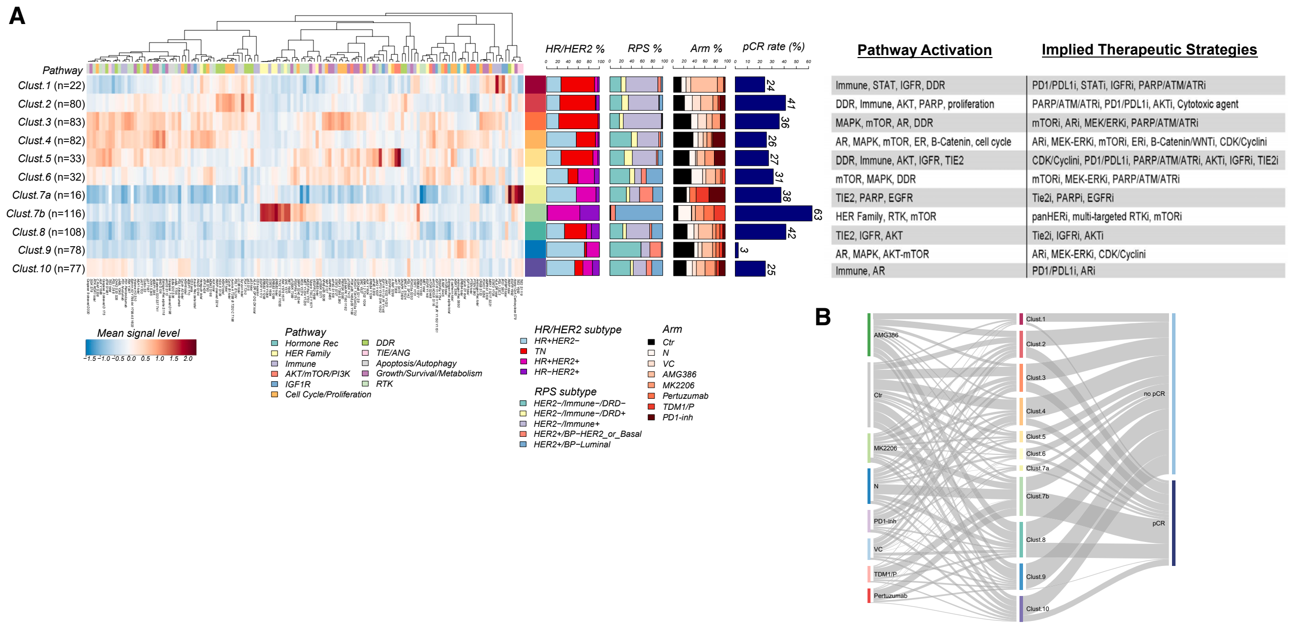

Figure 5. Protein/phosphoprotein signaling activation clusters linkage to sensitivity/resistance

(A) One-way hierarchical clustering map of protein/phosphoprotein analyte-mean signaling levels (columns) within each RPPA signaling cluster (rows). Heatmap color scale: red/white/blue, higher/intermediate/lower levels of expression.

(B) Sankey plot illustrating relationship of trial arms (left), RPPA signaling clusters (center), and pCR (right).

that are unlikely to respond to treatment but also pose little threat in the form of risk of distant metastasis.

Hazard ratio analysis of the DRFS interval within each of the 11 signaling clusters was performed on pCR versus non-pCR groups and the forest plots shown (Figure 6A; Table S4). Regardless of the underpinning signaling architecture, patients within every cluster who did not achieve pCR had a worse overall prognosis compared with those who achieved pCR, except for cluster 7a, which was the smallest cluster in the analysis (n = 16) (Figures 6A, 5A and S2). Kaplan-Meier DRFS curves for patients who did not achieve pCR within each of the 11 pathway signaling clusters are shown (Figure 6B.1).

Non-responding patients in cluster 9 have a DRFS rate of ~75% at 6 years with a DRFS hazard ratio of 0.26 compared with a population average DRFS rate of 95% at 6 years for patients who achieve pCR (Figures 6A and 6B.2). This difference in outcomes confirms an unmet need to identify effective treatments for this group. Within this group, higher levels of ERα, cyclin D1, and activated ROS were nominally associated with shorter DRFS (Figure 6C; Table S4). Thus, higher levels of these proteins both characterize cluster 9 relative to the other clusters (Figure 5), and trend toward association with poorer outcome in non-responders. These endpoints constitute potential drug targets, as do other signaling differences characterizing this cluster such as high activation/expression levels of ERK pathway signaling (i.e., ERK T202/Y204 and MSK1 S380) and IGFR signaling (i.e., IGFR Y1131/IR Y1146 and total IGFBP5) (Figure 5A).

Patients not achieving pCR in clusters 2 and 8 have the highest risk for distant recurrence of their disease. Non-responders in these clusters had 5-year DRFS of ~55% and ~52%, respectively (Figures 6B.3 and B.4). Decreased DRFS in cluster 2 non-responders was nominally associated with increased pan-RTK expression/activation (pFAK, pc-KIT, pALK, pAMPK, insulin receptor, ROR receptor), and in cluster 8 by a significantly decreased activation of pBRCA1 and nominally increased activation of SRC, TIE2, and A-RAF (Figure 6C; Table S4). Therapeutic strategies that target insulin/AMPK signaling and/or multi-TKI inhibitors that target ROS, ALK, KIT, FAK, TIE2, SRC, etc., may specifically target the tumor biology of these poorest-prognosis patients.

When we analyzed protein expression and signaling activation levels that associate globally with DRFS overall across all intrinsic subtypes in non-responding patients, we found that higher expression of cyclin B1, Ki67, and cleaved PARP D214, along with higher activation/phosphorylation of A-RAF S299, cKIT Y703, FAK Y397, and PDGFR Y754 nominally associated with poor DRFS overall (Figure 6C, top row). This more general result suggests therapeutics that target cKIT, FAK, PDGFR, and A-RAF, as well as therapeutics that target CDKs, and proliferation could be generally useful for rescue therapy in non-responders, although information on cluster membership may provide better guidance.

Fit-for-purpose protein/phosphoprotein signatures: HER2 activation response predictive signature as a case study

While our previously described RPS-5 subtyping schema was designed to maximize response rates (pCR) and is being prospectively validated in I-SPY 2.2, a genomic-based treatment strategy was not identified for the HER2−/Immune−/DRD− subtype, a cohort with very low response rates to all agent classes

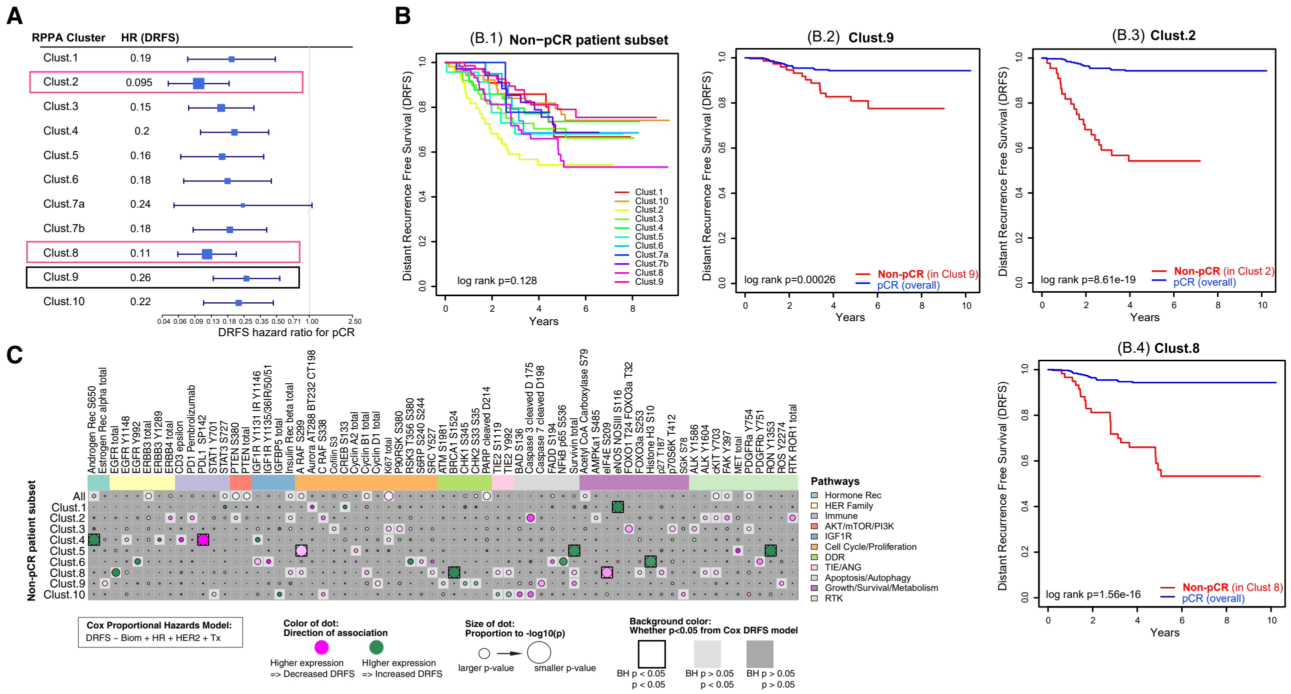

Figure 6. DRFS association within non-pCR patients by RPPA cluster

(A) Hazard ratio (HR) for DRFS for pCR by signaling cluster (box size, power; whiskers, 95% confidence interval (CI)).

(B) Kaplan-Maier plots of DRFS in the non-pCR patient subset for all 11 RPPA signaling clusters (B.1). (B.2–B.4) Kaplan-Meier plots of DRFS comparing patients achieving pCR across the whole population (blue curves), to non-pCR patients (red curves) in cluster 9 (B.2), cluster 2 (B.3), and cluster 8 (B.4).

(C) Dot plot demonstrating association of individual protein/phosphoprotein analytes (columns) with DRFS in each of the 11 RPPA signaling clusters (rows) within non-pCR patients. Analytes included are limited to those with significant or nominal association in at least one RPPA signaling cluster.

evaluated in I-SPY 2 to date (Figure 7[6]). Consequently, we sought to determine if our phosphoprotein/protein profiling could uncover new therapeutic options for patients with HER2−/Immune−/DRD− cancer. Our interest in developing more quantitative and accurate measurements of HER family proteins and pathway activity is based on our previous demonstration of measurable HER2-EGFR protein phosphorylation and downstream pathway activity in HER2− breast tumors in the I-SPY Trial.[24] We then extended this observation in the I-SPY 2 Trial where we demonstrated that HER2-EGFR co-activation/phosphorylation defined a signature, the HER2 activation response predictive signature (HARPS), which predicted pCR response in TN patients treated with neratinib.[14,24]

Based on this observation, we wanted to understand if HARPS could contribute useful information for determining the potential benefit of a HER2-targeted therapy in HER2−/low patient populations, especially within the HER2−/Immune−/DRD− RPS subtype. Because RPPA analysis is a semi-quantitative calibrated assay, we extrapolated our previously defined HARPS cut point[14] to the entire TNBC population in our study set (n = 252). This revealed that ∼40% of the TN patients in our entire dataset (n = 101) were HARPS+ (Figure 7A; Table S1). Eighty-two percent of TN/HARPS+ patients achieved pCR with N vs. 15% of TN/HARPS− (Figure 7B). We then evaluated the HARPS+ and HARPS− frequencies in the RPS-5 subtypes restricted to TN patients and found that 44% (28/63) of the TN/Immune−/DRD− patients are HARPS+ and thus potentially sensitive to HER2-directed therapeutics (Figure 7C). An added observation was that 82% of the TN/HARPS− patients achieved pCR with a PD1-inh vs. 56% of TN/HARPS+ (Figure 7B), thereby suggesting mechanistic links between low pERBB2/pEGFR and high immune activation, hinting at a potential HER-pathway biology that may increase the accuracy of predictive biomarkers for immunotherapy response.

DISCUSSION

Our recent companion publication focused on developing RPS based on genomic/transcriptomic data and introduced a subtyping schema to help prioritize treatments that are now standard of care.[6] Our analyses presented herein point to the complementary value of our signaling pathway activation mapping efforts with the RPS subtype schema. The HARPS signature could be used to "rescue" patients in the RPS HER2−/Immune−/DRD− subtype by directing TN patients with this signature that currently have no molecularly rationalized therapeutic options to HER2-directed or PD1-inh-based therapeutics. We have shown previously[24] that HARPS+ TN tumors have significantly increased HER2/EGFR-driven downstream signaling compared with HARPS− tumors. Moreover, we know from past work that HARPS+ TN patients are exceptional responders to the dual kinase inhibitor neratinib compared with HARPS− TN patients.[14] Taken together, these results strongly suggest that the co-activation of HER2/EGFR (HARPS+) in TN

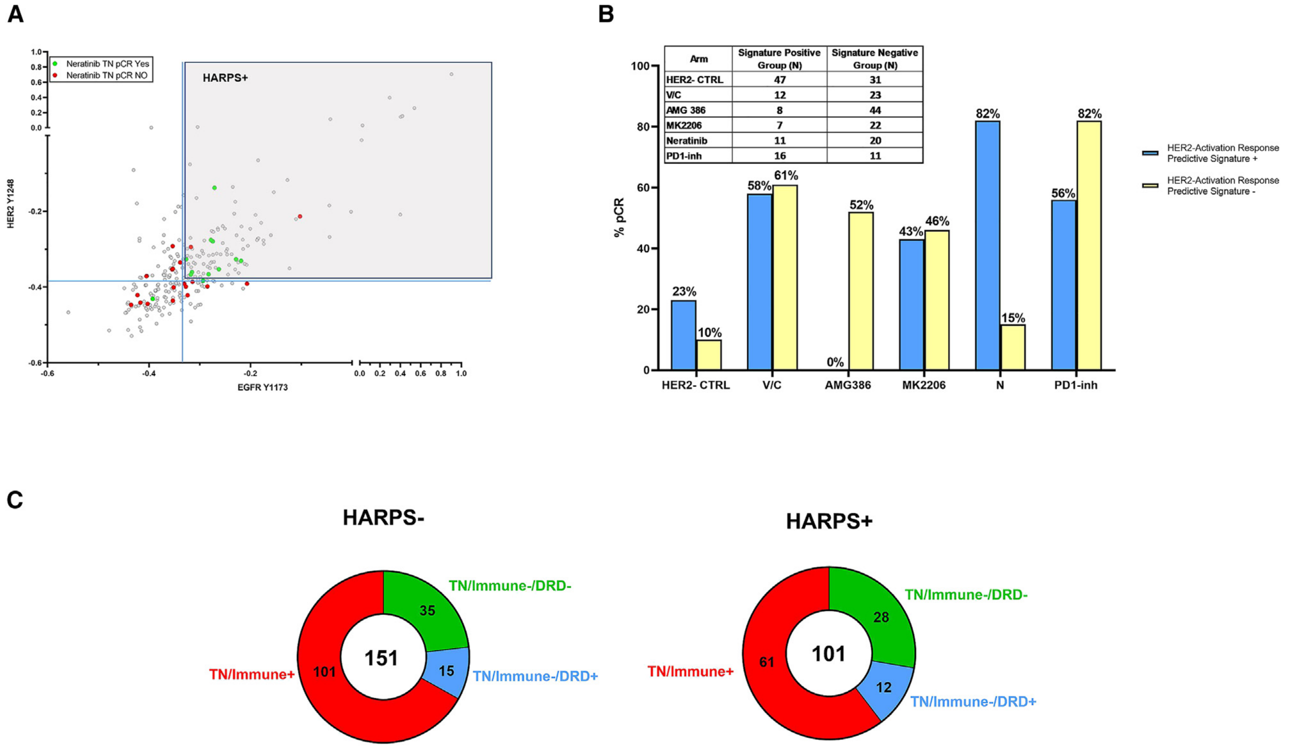

Figure 7. HARPS in TNBC

(A) Two-way scatterplot of HER2 Y1248 (y axis) and EGFR Y1173 (x axis) of TNBC patients (n = 252) with pre-defined neratinib response cut-point (blue solid line).[14]

(B) Response rates observed for TNBC patients whose tumors were HARPS+ or HARPS– across the six treatments arms for TNBC patients.

(C) Donut plots of the TN RPS signature distribution in both the HARPS+ and HARPS– cohorts. Numbers indicate individual patient numbers for each signature with overall number shown in the middle of circle.

patients transmits a biologically relevant and functional signal *in vivo* and that HARPS accurately predicts therapeutic response to a precision therapeutic modality. As we have also shown previously,[14] the phosphorylation levels of HER2 and EGFR that underpin HARPS can only be determined by measuring the phosphorylation state directly and are not predicted by measuring HER2 or EGFR at the mRNA level or total protein level and is not driven by HER2, EGFR, NRG1, AKT1, PIK3CA, and PTEN mutation/alteration/amplification.

We utilized laser microdissection to enrich the tumor epithelium across all 736 patient tumor samples prior to RPPA analysis. Previous proteomic studies relied on whole tissue lysates derived from samples with uncontrolled cellular input including mixtures of stroma, immune cell and tumor epithelium, fat, fibroblasts, etc., and did not adequately enrich for any cell type greater than 90%, which we have demonstrated greatly impacts the accuracy of protein and protein signaling data.[25–29] A further distinctive aspect of our analysis is that the protein expression and signaling activation data were derived from treatment-naive breast cancer tumor cells and that the study was undertaken to identify actionable protein targets that could be used to prioritize treatment strategies for future studies and in non-responding patient cohorts. These data and transcriptional profiling[6]—the I-SPY2-990 Data Resource compendium—are now publicly available to the research community. This resource may provide insights not only into mechanisms of *de novo* resistance and sensitivity extending beyond a genomics centered view but also generates a direct readout for prospective drug target selection and predictive fit-for-purpose signatures specific to MOAs of the therapeutic agents utilized.

The ability to correlate this very large set of data across multiple therapeutic arms with outcomes (pCR and DRFS) represents an opportunity to uncover intrinsic treatment-specific and global sensitivity and resistance predictive and prognostic biomarkers. We identified cyclin D1, ERα, and AR S650 as markers for global therapeutic resistance across all treatment arms and receptor subtypes. The finding that quantitative levels of ERα associated with resistance even in the HR− population is intriguing and points to the presence of a cohort of patients with low relative levels of ER but enough ER expression/signaling to drive a resistance phenotype to the drugs utilized in this population. Given that this cohort would generally not be provided ER-directed therapies, it is important to consider the investigation of hormonal-targeted therapies in this particular cohort of ER− patients who appear to have a measurable level of ER.

Phosphorylation of AR at S650 is known to mediate AR nuclear export and decrease transcriptional activity.[30–32] Our data suggest that utilization of therapeutic modulators targeting AR and cell-cycle-driven events may have clinical utility in patients whose tumors have signatures of global non-response. These

findings are important, as inclusion of new AR- and ER-targeting/endocrine-based approaches could be rationally considered in prospective I-SPY 2 arms to address resistance. Currently, AR inhibitors are being widely used to treat prostate cancer and are showing encouraging results in TNBC.[33] However, recent data suggest that ER can act as a “rheostat” and that targeting AR biology in the context of high relative ER co-expression may require the use of AR agonists in the setting where AR acts as a tumor suppressor compared with low ER expression where targeting AR with inhibitors would be biologically supported.[34]

Cyclin D1 is known to play a critical role in cell proliferation and is essential for the formation and maintenance of HER2+ tumors and response to ER-directed therapies.[35–37] Overexpression of cyclin D1 has been reported in invasive breast cancers and correlated with shorter disease-free survival dependent on molecular subtype.[38] Recently, it has been found that high expression of cyclin D1 and CDK4 mediate resistance to HER2-targeted therapies, but this acquired resistance can be disrupted by CDK4/6 inhibitors.[39] Our findings that increased cyclin D1 appears to be a universal resistance marker across all HR/HER2 subtypes suggest that cyclin D1 could be an actionable target using therapeutic approaches that promote tumor senescence or by modulating the cell cycle directly using CDK4/6 inhibitors (in the case of HER2+ tumors).[40]

The assembly of this dataset provided our first opportunity to examine baseline signaling architecture across the 736 patients we have analyzed by RPPA to date in the I-SPY 2 population. Unsupervised hierarchical clustering analysis of the study set yielded 11 protein signaling pathway-based clusters comprised of patients with common signaling activation patterns and revealed druggable targets that may not have been the target of the therapy they were randomized to receive in the trial.[10–14] Indeed, outside of cluster 7b, which comprised HER2+ and HER2− tumors with activated HER family signaling and was selected for HER2-targeted inhibitors, the patients in other clusters generally were not provided therapies that matched the underpinning pathway activation signatures measured. We identified patients in cluster 1 with elevated immune-related signatures that could have possibly benefited from immunotherapy agents (Figures 5A and S2, cluster 1). Cluster 5 was characterized by DRD pathway activation, elevated AKT phosphorylation, and TIE2 expression, which suggests that more patients in this cluster might achieve pCR by including AKT inhibitors in their treatment regimen in addition to immunotherapy and/or TIE2 inhibitors (Figures 5A and S2,cluster 5). Likewise, cluster 7a displayed elevated levels of TIE2 S1119, TIE2 Y992, but most patients were randomized to receive HER2-targeted therapy instead of TIE2 inhibitors (Figures 5A and S2,cluster 7a). We have previously shown that elevation of pTIE2 correlates with response to AMG386, a TIE/ANG-targeted agent[6,23] and those with activated AKT signaling pathway associate with response to MK2206 and ipatasertib treatment in HER2+ and HER2− populations.[12,13] We observed unexpected differences in JAK/TYK-STAT pathway immune signaling proteins characterizing “immune high” HER2+ and TNBC populations. These results suggest a difference in tumor immune signaling between HER2+ and TNBC cancers that could point to a subtype-specific immunotherapy response-predictive biomarker. However, this will require validation and interrogation of study sets that include HER2+ patients neoadjuvantly treated with immunotherapeutics. Because our analysis in this paper is focused on LCM-enriched tumor epithelium and not the stroma/immune cells within the tumor microenvironment, these specific differences in tumor epithelium JAK/TYK-STAT signaling observed could arise from paracrine/autocrine changes in the tumor epithelium signaling resulting from interactions with the stroma/immune cells.

From a prognostic standpoint, we found that non-responding patients in cluster 9, who mostly (97%) do not achieve pCR, also have a comparatively poor overall DRFS (approximately 75% at 9 years). These patients were characterized by elevated levels of cyclin D1, ERα, and AR S650 (the global resistance signature) along with relatively high expression/activation of members of the ERK pathway and IGFR signaling pathway (Figure 5A). These results suggest that, in addition to therapeutics that could be used to overcome global resistance by targeting cyclin D1, ERα, and pAR, patients within cluster 9 may have responded positively to agents targeting MEK/ERK and/or IGFR signaling instead of the standard of care chemotherapy most of them received. As discussed previously, targeting cyclin D1 via CDK4/6 inhibition may prevent the phosphorylation of retinoblastoma family proteins (RB), which regulate the G1-S-phase progression of the cell cycle.[41] In a CDK-independent environment, inhibiting the DNA-binding activity of cyclin D1 and HDACs may interfere with gene expression, cell proliferation, and differentiation of tumor cells.[42] Our results show that developing new treatment approaches for patients whose tumors display this characteristic protein signaling signature as well as others with a “global resistance phenotype” marked by high cyclin D1 is urgent.

In the same manner, we found that non-responding patients in cluster 2 and cluster 8 have extremely poor long-term prognosis and, therefore, achieving pCR for these patients is critically important. Potential clinically actionable targets include several RTKs and downstream signaling molecules (Figure 5A). In cluster 8, low levels of activated BRCA1 S1524 and increased activation of the mTOR kinase substrate, eIF4E S209, significantly associated (BH $p < 0.05$) with lower DRFS in non-pCR patients (Figure 6C), while higher levels of pALK, pcKIT, and pFAK were among the phosphoproteins nominally associated with lower DRFS in non-pCR patients in cluster 2. These data provide a rationale for considering mTOR inhibitors and broad multi-targeted TKIs for the ongoing I-SPY 2.2 block design to target these pathways in patients with these tumor signatures who do not achieve pCR and have worse DRFS. It is important to note that, despite the relatively high pCR rates of clusters 2 and 8, nearly half of the patients in both clusters received standard of care chemotherapy or AMG386 (Figures 5B and S2) but, based on their tumor protein signaling architecture, these patients hypothetically might have responded to PD1-inh, MK2206, or other inhibitors that target their activated signaling networks. In the future, functional pathway activation mapping could be used to identify patients who are destined for non-response to the targeted agents used in the I-SPY 2 Trial to date and to select alternative agents that target their individually activated pathways.

CellPress
OPEN ACCESS

In addition to targeting intrinsic, subtype-independent resistance mechanisms underpinned by ERα, pAR, and cyclin D1, we found druggable targets that associate with specific pathway-driven signatures (Figure 5A) and/or poor prognosis/DRFS (Figure 6A) including CDK/cyclin B, cKIT, FAK, PDGFR, and A-RAF signaling. Because achieving pCR associates so strongly with overall DRFS in our dataset, it is not surprising that we did not observe any protein/phosphoprotein significantly associated with poor overall DRFS in subjects who reached pCR, as the number of patients who achieved a pCR have >95% DRFS (median follow-up 4.5 years). The finding that specific druggable protein targets such as ERα, cyclin D1, and pAR were elevated as a global resistance signature could help prioritize agents that directly target those proteins/pathways as a molecularly informed regimen for future clinical trial considerations to overcome inherent resistance. Moreover, targeted agents that inhibit protein expression/activity of proteins and pathways identified in our signaling defined clusters (Figure 5A), as well as those associated with poor DRFS, could be prioritized and considered in next iterations of clinical trials such as I-SPY 2.2. Many of the proteins/phosphoproteins that we found to be significantly expressed/activated as markers of global resistance, non-pCR, and/or poor DRFS correlate with cell culture-based drug sensitivity[43,44] as well as clinical response[13,23,40,45–47] to drugs that target those specific proteins (HER2, EGFR, TIE2, AR, AKT, mTOR, etc.). These data support the functional significance of our findings and provide justification for further exploration of the implied therapeutic strategies in prospective trials. We identified that activation of PLK1/FADD-based DDR signaling pathway activation predicted non-response in HR−/HER2+ patients who received T-DM1/P therapy (Figure S1D). This finding is corroborated by previous cell line-based studies that identified the same pathway as an important resistance mechanism to TDM-1 in HER2+ tumors, which could be reversed through the addition of the PLK1 inhibitor volasertib *in vitro*.[48] Consequently, our results suggest that combining a PLK1 inhibitor with a HER2 inhibitor in PLK1-activated HR–HER2+ tumors or TDM-1 refractory disease could be synergistic and a molecularly rationalized approach for future I-SPY 2 arms.

While the RPS-5 classification schema reported in Wolf et al. provides transcriptomic-based response prediction to broad classes of therapeutic agents (i.e., immunotherapy and DNA-damaging agents), the work presented in this article identifies specific protein/phosphoprotein fit-for-purpose actionable markers tied to the MOA of the drug itself, such as HARPS, that could be used in synergy with the HER2−/Immune−/DRD− RPS subtype. Under the current RPS subtype schema, patients with this transcriptomic signature have no molecularly predicted agents to consider; however, our results reveal that nearly 45% of these patients are HARPS+ and are predicted to respond to HER2-targeting agents such as neratinib. Overall, there were no apparent associations of any RPS-5 subtype with protein signaling-based clusters with the exception of cluster 7b and cluster 3 (Figure 5A). Cluster 7b is comprised nearly exclusively of HER2+ tumors with HER2/HER family pathway activation and comprised of the RPS HER2+/Luminal subtype. Cluster 3 is comprised nearly exclusively of TN tumors with increased PDL1 expression/immune signaling (along with ERK and mTOR pathway activation) and largely comprised of the RPS HER2−/Immune+ subtype.

Ultimately, it is our hope that phosphoprotein/protein-based fit-for-purpose protein/phosphoprotein markers such as HARPS will synergize with the RPS-based subtyping schema as we continue to refine and adapt I-SPY 2 by learning from the tumor biology and outcomes observed. Protein/phosphoprotein-based drug target activation analysis provides a biochemical means to prioritize and select therapeutic drug classes based on pathway activation in tumors from patients who do not achieve pCR and fit within the context of the current RPS-5 prospective schema implemented for I-SPY 2.2, especially for the HER2−/Immune−/DRD− subtype. We found that the activation levels of drug targets provide useful, actionable information about drug response that is often independent of genomic alteration, HR/HER2 status, PAM50, or other molecular subtyping criteria.[10,12–14,24] The prospective application of HARPS as a fit-for-purpose biomarker, if validated in larger studies, raises the intriguing future vision where tumors from all TN patients would be analyzed upfront for HARPS status and all patients with HARPS+ tumors would receive a HER2-directed therapy (ADC or TKI) and patients with HARPS− tumors would receive an anti PDL1/PD1 therapy. The overall predicted pCR rate for TNBC could approach ∼80% with just these two therapeutic classes and one phosphoprotein-based fit-for-purpose biomarker signature. The potential use of HARPS in prospective TN patient stratification could synergize well with the recent approval of trastuzumab deruxtecan in the HER2 LOW setting (IHC 1+ and IHC 2+/FISH−) by providing a better understanding of the potential for therapeutic efficacy of HER2-targeting agents (ADC and TKI) in patients with HER2 IHC 0 "ultra-low" disease, as well as helping to refine the lower limit of the HER2-LOW designation overall.[49–51]

Our ability to analyze the RPPA-generated protein signaling data across arms, agents, and subtypes provides an important opportunity to analyze and uncover protein expression and signaling activation biomarkers/signatures from a large pool of patient tumor samples that associate with global non-response/resistance as well as indicate possible new therapeutic targets and strategies for therapeutic prioritization. There remains a significant number of breast cancer patients who do not respond to existing treatments. Therefore, identifying new classification schemas based on global RPS signatures, tumor biology, protein expression/activation profiling, and fit-for-purpose signatures such as HARPS that form the basis of more effective therapeutic strategies and patient stratification, remain an urgent priority.

Limitations of this study

The adaptive design used to randomize patients in the I-SPY 2 Trial can result in low patient numbers in specific agent arms and within subtype signatures, which presents clear limitations for the biomarker discovery work presented here. The overall data are hypothesis generating and further research is necessary to validate our findings. Because I-SPY 2 currently excludes patients who are not MammaPrint high risk, the trial is not a natural history cohort and this is likely to introduce bias in the survival outcomes seen. Our study focuses on protein expression and

signaling activation analysis in LCM-enriched tumor epithelium and not the stromal/immune compartments of the tumor microenvironment; thus, outcome correlations and clinical measurements are limited to tumor epithelium biology and do not account relevant aspects of stroma/immune tumor biology. Consequently, the generalizability of the biomarker findings described will require further validation in other settings.

STAR★METHODS

Detailed methods are provided in the online version of this paper and include the following:

- KEY RESOURCES TABLE
- RESOURCE AVAILABILITY
  - Lead contact
  - Materials availability
  - Data and code availability
- EXPERIMENTAL MODEL AND STUDY PARTICIPANT DETAILS
  - I-SPY 2 TRIAL overview
  - Trial design
  - Eligibility
  - Treatment
  - Trial Oversight
- METHOD DETAILS
  - Pretreatment biopsy microdissection and molecular profiling
  - RPPA data analysis
- QUANTIFICATION AND STATISTICAL ANALYSIS
  - Statistical analysis of continuous RPPA biomarkers
- ADDITIONAL RESOURCES

SUPPLEMENTAL INFORMATION

Supplemental information can be found online at https://doi.org/10.1016/j.xcrm.2023.101312.

ACKNOWLEDGMENTS

This work was supported by funding from Quantum Leap Healthcare Collaborative, FNIH, NIH/NCI I-SPY 2+ (grant PO1-CA210961), NIH/NCI Imaging (grant 28XS197 P-0518835), NIH/NCI CCMI (grant U54CA209891), NIH/NCI CCSG (grant P30- CA82103), NIH/NHGRI Big Data (grant U54-HG007990), Safeway—an Albertsons Company, William K. Bowes, Jr., Foundation, Breast Cancer Research Foundation (BCRF-20-165), UCSF, GMU, Gateway for Cancer Research (grants G-16-900 and G-20-600), SideOut Foundation, the Biomarkers Consortium, Salesforce, OpenClinica, Formedix, Hologic Inc., TGen, CCS Associates, Berry Consultants, Breast Cancer Research – Atwater Trust, Stand up to Cancer, California Breast Cancer Research Program, and Give Breast Cancer the Boot. Support was also provided by Angela and Shu Kai Chan Chair in Cancer Research (L.J.v.t.V.), IQVIA, Genentech, Amgen, Pfizer, Merck, Seattle Genetics, Daiichi Sankyo, AstraZeneca, Dynavax Technologies, Puma Biotechnology, AbbVie, Madrigal Pharmaceuticals (formerly Synta), Plexxikon, Regeneron, and Agendia. Sincere thanks to our DSMB, Independent Agent Selection Committee, and Biomarker Working Group and to our patients, advocates, and investigators.

AUTHOR CONTRIBUTIONS

R.I.G., J.W., and D.M.W. contributed equally to this study. R.I.G., J.W., D.M.W., E.F.P., and L.J.v.V. designed the study, interpreted the data, and prepared and reviewed the manuscript along with L.J.E. and C.Y. D.M.W. analyzed the data, with assistance from M.J.M. and A.B. R.I.G., J.W., and E.F.P. generated the RPPA data. L.B.-S. leads the I-SPY lab, overseeing molecular assays. N.O. and J.B.M. managed the data and manuscript submission. G.L.H. manages the Biomarker Working Group led by L.J.v.t.V., with members D.M.W., C.Y., J.W., L.B.-S., M.J.M., A.B., M.C.L., J.-P.C., M.J.C., and W.F.S. L.S. manages the I-SPY 2/2.2 P01; J.P. is a patient advocate; and M.C.L., P.R.P., W.F.S., H.S.R., C.I., A.M.D., and D.Y. are I-SPY 2 working group leads. S.M.A. managed I-SPY Trials operations, R.L. is trial statistician, and L.J.E. and N.H. are the principal investigators of I-SPY 2. I-SPY 2 Trial Investigators and Biomarker and other Working Group members participate in all aspects of the trial and contribute to its success. All authors participated in manuscript preparation and review.

DECLARATION OF INTERESTS

J.W. reports honoraria from DAVA Oncology, consults for Baylor College of Medicine, has ownership in Theralink, and is co-inventor of RPPA technology and p-HER2 and -EGFR response predictors with filed patents. C.Y. consults for NantOmics, LLC. M.C.L. reports support from Eisai, Genentech, GRAIL, Menarini Silicon Biosystems, Merck, Novartis, Seattle Genetics, and Tesaro. W.F.S. is a co-founder of Delphi Diagnostics and co-inventor/patent holder for a free residual cancer burden calculator, holds shares in IONIS Pharmaceuticals and Eiger Biopharmaceuticals, and is an unpaid advisor/steering committee member for Roche trials. H.S.R. reports support from Pfizer, Merck, Novartis, Lilly, Roche, Daiichi, Seattle Genetics, Macrogenics, Sermonix, Boehringer Ingelheim, Polyphor, AstraZeneca, Astellas, and Gilead; honoraria from Puma Biotechnology, Samsung, Chugai, Blueprint, and NAPO; and travel support from GE Healthcare. C.I. consults for Seattle Genetics, Genentech, AstraZeneca, Novartis, PUMA, Pfizer, and Esai. A.M.D. reports support from Novartis, Pfizer, Genentech, Calithera, and Menarini. D.Y. receives unrelated support from Boehringer Ingleheim and consults with Martell Diagnostics unrelated to this topic. P.R.P. reports leadership and stock in Immunonet BioSciences and honoraria from ASCO, Dava Oncology, OncLive (Courses), and Frontiers (Editorship); consults for Personalized Cancer Therapy, Immunonet BioSciences, Sirtex, CARIS Lifesciences, OncoPlex Diagnostics, Pfizer, Heron, Puma, AbbVie, BOLT, and SEAGEN; and is an occasional speaker for Genentech and Roche. G.L.H. is a partner and holds stock (<1%) in NanoString, Moderna, Gilead Sciences, and Exact Sciences. L.J.E. is an unpaid member of the board of directors of Quantum Leap Healthcare Collaborative, received grant support for the I-SPY 2 trial, is on the Blue Cross/Blue Shield Medical Advisory Panel and receives support for time and travel, and receives unrelated research support from Merck. L.J.v.V. is a part-time employee and stockholder of Agendia, NV. E.F.P. reports leadership, stock/ownership, and consulting/advisory and travel funds from Theralink Technologies, Inc., Perthera, Inc., and Ceres Nanosciences, Inc.; support from Ceres Nanosciences, GlaxoSmithKline, Abbvie, Symphogen, Deciphera Pharmaceuticals, Inc, Springworks Therapeutics, Inc, Mirati, Inc. and Genentech; patents/royalties from NIH; and patents/roylaties for anti-HER2/EGFR and anti-mTOR response predictors.

INCLUSION AND DIVERSITY

We support inclusive, diverse, and equitable conduct of research.

Received: December 7, 2022
Revised: July 3, 2023
Accepted: November 14, 2023
Published: December 11, 2023

REFERENCES

1. DeSantis, C.E., Ma, J., Gaudet, M.M., Newman, L.A., Miller, K.D., Goding Sauer, A., Jemal, A., and Siegel, R.L. (2019). Breast cancer statistics, 2019. CA. Cancer J. Clin. *69*, 438–451.

2. Siegel, R.L., Miller, K.D., Wagle, N.S., and Jemal, A. (2023). Cancer statistics, 2023. CA. Cancer J. Clin. *73*, 17–48.
3. Freitas, A.J.A.d., Causin, R.L., Varuzza, M.B., Hidalgo Filho, C.M.T., Silva, V.D.d., Souza, C.d.P., and Marques, M.M.C. (2021). Molecular Biomarkers Predict Pathological Complete Response of Neoadjuvant Chemotherapy in Breast Cancer Patients: Review. Cancers *13*, 5477.
4. Mueller, C., Haymond, A., Davis, J.B., Williams, A., and Espina, V. (2018). Protein biomarkers for subtyping breast cancer and implications for future research. Expert Rev. Proteomics *15*, 131–152.
5. I-SPY2 Trial Consortium; Yee, D., DeMichele, A.M., Yau, C., Isaacs, C., Symmans, W.F., Albain, K.S., Chen, Y.Y., Krings, G., Wei, S., et al. (2020). Association of Event-Free and Distant Recurrence–Free Survival With Individual-Level Pathologic Complete Response in Neoadjuvant Treatment of Stages 2 and 3 Breast Cancer: Three-Year Follow-up Analysis for the I-SPY 2 Adaptively Randomized Clinical Trial. JAMA Oncol. *6*, 1355–1362.
6. Wolf, D.M., Yau, C., Wulfkuhle, J., Brown-Swigart, L., Gallagher, R.I., Lee, P.R.E., Zhu, Z., Magbanua, M.J., Sayaman, R., O'Grady, N., et al. (2022). Redefining breast cancer subtypes to guide treatment prioritization and maximize response: Predictive biomarkers across 10 cancer therapies. Cancer Cell *40*, 609–623.e6.
7. Spring, L.M., Fell, G., Arfe, A., Sharma, C., Greenup, R., Reynolds, K.L., Smith, B.L., Alexander, B., Moy, B., Isakoff, S.J., et al. (2020). Pathologic Complete Response after Neoadjuvant Chemotherapy and Impact on Breast Cancer Recurrence and Survival: A Comprehensive Meta-analysis. Clin. Cancer Res. *26*, 2838–2848.
8. Yau, C., Osdoit, M., van der Noordaa, M., Shad, S., Wei, J., de Croze, D., Hamy, A.-S., Laé, M., Reyal, F., Sonke, G.S., et al. (2022). Residual cancer burden after neoadjuvant chemotherapy and long-term survival outcomes in breast cancer: a multicentre pooled analysis of 5161 patients. Lancet Oncol. *23*, 149–160.
9. Loebke, C., Sueltmann, H., Schmidt, C., Henjes, F., Wiemann, S., Poustka, A., and Korf, U. (2007). Infrared-based protein detection arrays for quantitative proteomics. Proteomics *7*, 558–564.
10. Clark, A.S., Yau, C., Wolf, D.M., Petricoin, E.F., van 't Veer, L.J., Yee, D., Moulder, S.L., Wallace, A.M., Chien, A.J., Isaacs, C., et al. (2021). Neoadjuvant T-DM1/pertuzumab and paclitaxel/trastuzumab/pertuzumab for HER2+ breast cancer in the adaptively randomized I-SPY 2 trial. Nat. Commun. *12*, 6428.
11. Gonzalez-Ericsson, P.I., Wulfkhule, J.D., Gallagher, R.I., Sun, X., Axelrod, M.L., Sheng, Q., Luo, N., Gomez, H., Sanchez, V., Sanders, M., et al. (2021). Tumor-Specific Major Histocompatibility-II Expression Predicts Benefit to Anti–PD-1/L1 Therapy in Patients With HER2-Negative Primary Breast Cancer. Clin. Cancer Res. *27*, 5299–5306.
12. Shi, Z., Wulfkuhle, J., Nowicka, M., Gallagher, R.I., Saura, C., Nuciforo, P.G., Calvo, I., Andersen, J., Passos-Coelho, J.L., Gil-Gil, M.J., et al. (2022). Functional Mapping of AKT Signaling and Biomarkers of Response From the FAIRLANE Trial of Neoadjuvant Ipatasertib Plus Paclitaxel for Triple-Negative Breast Cancer. Clin. Cancer Res. *28*, 993–1003.
13. Wolf, D.M., Yau, C., Wulfkuhle, J., Brown-Swigart, L., Gallagher, R.I., Magbanua, M.J.M., O'Grady, N., O'Grady, N., I-SPY 2 TRIAL Investigators; and Asare, S., et al. (2020). Mechanism of action biomarkers predicting response to AKT inhibition in the I-SPY 2 breast cancer trial. NPJ Breast Cancer *6*, 48.
14. Wulfkuhle, J.D., Yau, C., Wolf, D.M., Vis, D.J., Gallagher, R.I., Brown-Swigart, L., Hirst, G., Voest, E.E., DeMichele, A., Hylton, N., et al. (2018). Evaluation of the HER/PI3K/AKT Family Signaling Network as a Predictive Biomarker of Pathologic Complete Response for Patients With Breast Cancer Treated With Neratinib in the I-SPY 2 TRIAL. JCO Precis. Oncol. *2*, 1–20.
15. Shenoy, A., Belugali Nataraj, N., Perry, G., Loayza Puch, F., Nagel, R., Marin, I., Balint, N., Bossel, N., Pavlovsky, A., Barshack, I., et al. (2020). Proteomic patterns associated with response to breast cancer neoadjuvant treatment. Mol. Syst. Biol. *16*, e9443.
16. Tang, X., Thompson, K.J., Kalari, K.R., Sinnwell, J.P., Suman, V.J., Vedell, P.T., McLaughlin, S.A., Northfelt, D.W., Aspitia, A.M., Gray, R.J., et al. (2023). Integration of multiomics data shows down regulation of mismatch repair and tubulin pathways in triple-negative chemotherapy-resistant breast tumors. Breast Cancer Res. *25*, 57.
17. Cerbelli, B., Pernazza, A., Botticelli, A., Fortunato, L., Monti, M., Sciattella, P., Campagna, D., Mazzuca, F., Mauri, M., Naso, G., et al. (2017). PD-L1 Expression in TNBC: A Predictive Biomarker of Response to Neoadjuvant Chemotherapy? BioMed Res. Int. *2017*, 1750925–1750927.
18. Alves, W.E.F.M., Bonatelli, M., Dufloth, R., Kerr, L.M., Carrara, G.F.A., Da Costa, R.F.A., Scapulatempo-Neto, C., Tiezzi, D., Da Costa Vieira, R.A., and Pinheiro, C. (2019). CAIX is a predictor of pathological complete response and is associated with higher survival in locally advanced breast cancer submitted to neoadjuvant chemotherapy. BMC Cancer *19*, 1173.
19. Yoshioka, T., Hosoda, M., Yamamoto, M., Taguchi, K., Hatanaka, K.C., Takakuwa, E., Hatanaka, Y., Matsuno, Y., and Yamashita, H. (2015). Prognostic significance of pathologic complete response and Ki67 expression after neoadjuvant chemotherapy in breast cancer. Breast Cancer *22*, 185–191.
20. Owen, K.L., Brockwell, N.K., and Parker, B.S. (2019). JAK-STAT Signaling: A Double-Edged Sword of Immune Regulation and Cancer Progression. Cancers *11*, 2002.
21. Wulfkuhle, J.D., Yau, C., Wolf, D.M., Gallagher, R.I., Brown Swigart, L., Hirst, G.L., Campbell, M., Nanda, R., Liu, M.C., Pusztai, L., et al. (2019). Quantitative MHC II protein expression levels in tumor epithelium to predict response to the PD1 inhibitor pembrolizumab in the I-SPY 2 Trial. J. Clin. Orthod. *37*, 2631.
22. Binnewies, M., Mujal, A.M., Pollack, J.L., Combes, A.J., Hardison, E.A., Barry, K.C., Tsui, J., Ruhland, M.K., Kersten, K., Abushawish, M.A., et al. (2019). Unleashing Type-2 Dendritic Cells to Drive Protective Antitumor CD4+ T Cell Immunity. Cell *177*, 556–571.e16.
23. Gallagher, R.I., Wulfkuhle, J.D., Yau, C., Wolf, D.M., Brown Swigart, L., Hirst, G.L., Esserman, L., Berry, D.A., van 't Veer, L., and Petricoin, E. (2018). Association of activation levels of TIE2 with response to the angiogenesis inhibitor trebananib in HER2+ patients in the I-SPY 2 trial. J. Clin. Orthod. *36*, 12103.
24. Wulfkuhle, J.D., Berg, D., Wolff, C., Langer, R., Tran, K., Illi, J., Espina, V., Pierobon, M., Deng, J., DeMichele, A., et al. (2012). Molecular analysis of HER2 signaling in human breast cancer by functional protein pathway activation mapping. Clin. Cancer Res. *18*, 6426–6435.
25. Baldelli, E., Haura, E.B., Crinò, L., Cress, D.W., Ludovini, V., Schabath, M.B., Liotta, L.A., Petricoin, E.F., and Pierobon, M. (2015). Impact of upfront cellular enrichment by laser capture microdissection on protein and phosphoprotein drug target signaling activation measurements in human lung cancer: Implications for personalized medicine. Proteomics. Clin. Appl. *9*, 928–937.
26. Hunt, A.L., Pierobon, M., Baldelli, E., Oliver, J., Mitchell, D., Gist, G., Bateman, N.W., Larry Maxwell, G., Petricoin, E.F., and Conrads, T.P. (2020). The impact of ultraviolet- and infrared-based laser microdissection technology on phosphoprotein detection in the laser microdissection-reverse phase protein array workflow. Clin Proteom *17*, 9.
27. Liotta, L.A., Pappalardo, P.A., Carpino, A., Haymond, A., Howard, M., Espina, V., Wulfkuhle, J., and Petricoin, E. (2021). Laser Capture Proteomics: spatial tissue molecular profiling from the bench to personalized medicine. Expert Rev. Proteomics *18*, 845–861.
28. Mueller, C., Davis, J.B., and Liotta, L.A. (2019). Combining the "Sibling Technologies" of Laser Capture Microdissection and Reverse Phase Protein Microarrays. In Reverse Phase Protein Arrays Advances in Experimental Medicine and Biology, T. Yamada, S.S. Nishizuka, G.B. Mills, and L.A. Liotta, eds. (Springer Singapore), pp. 95–111.
29. Li, Q.K., Hu, Y., Chen, L., Schnaubelt, M., Cui Zhou, D., Li, Y., Lu, R.J.-H., Thiagarajan, M., Hostetter, G., Newton, C.J., et al. (2022). Neoplastic cell enrichment of tumor tissues using coring and laser microdissection for

proteomic and genomic analyses of pancreatic ductal adenocarcinoma. Clin Proteom *19*, 36.

30. Chen, S., Kesler, C.T., Paschal, B.M., and Balk, S.P. (2009). Androgen Receptor Phosphorylation and Activity Are Regulated by an Association with Protein Phosphatase 1. J. Biol. Chem. *284*, 25576–25584.
31. Gioeli, D., Black, B.E., Gordon, V., Spencer, A., Kesler, C.T., Eblen, S.T., Paschal, B.M., and Weber, M.J. (2006). Stress Kinase Signaling Regulates Androgen Receptor Phosphorylation, Transcription, and Localization. Mol. Endocrinol. *20*, 503–515.
32. Gordon, M.A., D'Amato, N.C., Gu, H., Babbs, B., Wulfkuhle, J., Petricoin, E.F., Gallagher, I., Dong, T., Torkko, K., Liu, B., et al. (2017). Synergy between Androgen Receptor Antagonism and Inhibition of mTOR and HER2 in Breast Cancer. Mol. Cancer Ther. *16*, 1389–1400.
33. Anestis, A., Zoi, I., Papavassiliou, A.G., and Karamouzis, M.V. (2020). Androgen Receptor in Breast Cancer—Clinical and Preclinical Research Insights. Molecules *25*, 358.
34. Hickey, T.E., Selth, L.A., Chia, K.M., Laven-Law, G., Milioli, H.H., Roden, D., Jindal, S., Hui, M., Finlay-Schultz, J., Ebrahimie, E., et al. (2021). The androgen receptor is a tumor suppressor in estrogen receptor–positive breast cancer. Nat. Med. *27*, 310–320.
35. Choi, Y.J., Li, X., Hydbring, P., Sanda, T., Stefano, J., Christie, A.L., Signoretti, S., Look, A.T., Kung, A.L., von Boehmer, H., and Sicinski, P. (2012). The requirement for cyclin D function in tumor maintenance. Cancer Cell *22*, 438–451.
36. Prat, A., and Baselga, J. (2008). The role of hormonal therapy in the management of hormonal-receptor-positive breast cancer with co-expression of HER2. Nat. Clin. Pract. Oncol. *5*, 531–542.
37. Giuliano, M., Hu, H., Wang, Y.-C., Fu, X., Nardone, A., Herrera, S., Mao, S., Contreras, A., Gutierrez, C., Wang, T., et al. (2015). Upregulation of ER Signaling as an Adaptive Mechanism of Cell Survival in HER2-Positive Breast Tumors Treated with Anti-HER2 Therapy. Clin. Cancer Res. *21*, 3995–4003.
38. Ortiz, A.B., Garcia, D., Vicente, Y., Palka, M., Bellas, C., and Martin, P. (2017). Prognostic significance of cyclin D1 protein expression and gene amplification in invasive breast carcinoma. PLoS One *12*, e0188068.
39. Goel, S., Wang, Q., Watt, A.C., Tolaney, S.M., Dillon, D.A., Li, W., Ramm, S., Palmer, A.C., Yuzugullu, H., Varadan, V., et al. (2016). Overcoming Therapeutic Resistance in HER2-Positive Breast Cancers with CDK4/6 Inhibitors. Cancer Cell *29*, 255–269.
40. Kato, S., Porter, R., Okamura, R., Lee, S., Zelichov, O., Tarcic, G., Vidne, M., and Kurzrock, R. (2021). Functional measurement of mitogen-activated protein kinase pathway activation predicts responsiveness of RAS-mutant cancers to MEK inhibitors. Eur. J. Cancer *149*, 184–192.
41. Malumbres, M., and Barbacid, M. (2009). Cell cycle, CDKs and cancer: a changing paradigm. Nat. Rev. Cancer *9*, 153–166.
42. Inoue, K., and Fry, E.A. (2015). Aberrant expression of cyclin D1 in cancer. Sign. Transduct. Insights *4*, 1–13.
43. Boyd, Z.S., Wu, Q.J., O'Brien, C., Spoerke, J., Savage, H., Fielder, P.J., Amler, L., Yan, Y., and Lackner, M.R. (2008). Proteomic analysis of breast cancer molecular subtypes and biomarkers of response to targeted kinase inhibitors using reverse-phase protein microarrays. Mol. Cancer Ther. *7*, 3695–3706.
44. Costello, J.C., Heiser, L.M., Georgii, E., Gönen, M., Menden, M.P., Wang, N.J., Bansal, M., Ammad-ud-din, M., Hintsanen, P., Khan, S.A., et al. (2014). A community effort to assess and improve drug sensitivity prediction algorithms. Nat. Biotechnol. *32*, 1202–1212.
45. Giuliani, R., Durbecq, V., Di Leo, A., Paesmans, M., Larsimont, D., Leroy, J.-Y., Borms, M., Vindevoghel, A., Jerusalem, G., D'Hondt, V., et al. (2007). Phosphorylated HER-2 tyrosine kinase and Her-2/neu gene amplification as predictive factors of response to trastuzumab in patients with HER-2 overexpressing metastatic breast cancer (MBC). Eur. J. Cancer *43*, 725–735.
46. Serrano-Heras, G., Cuenca-López, M.D., Montero, J.C., Corrales-Sanchez, V., Morales, J.C., Núñez, L.E., Morís, F., Pandiella, A., and Ocaña, A. (2015). Phospho-kinase profile of colorectal tumors guides in the selection of multi-kinase inhibitors. Oncotarget *6*, 31272–31283.
47. Wang, F., Wang, S., Wang, Z., Duan, J., An, T., Zhao, J., Bai, H., and Wang, J.; Key Laboratory of Carcinogenesis and Translational Research Ministry of Education (2012). Phosphorylated EGFR expression may predict outcome of EGFR-TKIs therapy for the advanced NSCLC patients with wild-type EGFR. J. Exp. Clin. Cancer Res. *31*, 65.
48. Saatci, Ö., Borgoni, S., Akbulut, Ö., Durmuş, S., Raza, U., Eyüpoğlu, E., Alkan, C., Akyol, A., Kütük, Ö., Wiemann, S., and Şahin, Ö. (2018). Targeting PLK1 overcomes T-DM1 resistance via CDK1-dependent phosphorylation and inactivation of Bcl-2/xL in HER2-positive breast cancer. Oncogene *37*, 2251–2269.
49. Viale, G., Basik, M., Niikura, N., Tokunaga, E., Brucker, S., Penault-Llorca, F., Hayashi, N., Sohn, J., Teixeira De Sousa, R., Brufsky, A.M., et al. (2023). Retrospective study to estimate the prevalence and describe the clinicopathological characteristics, treatments received, and outcomes of HER2-low breast cancer. ESMO Open *8*, 101615.
50. Mosele, F., Deluche, E., Lusque, A., Le Bescond, L., Filleron, T., Pradat, Y., Ducoulombier, A., Pistilli, B., Bachelot, T., Viret, F., et al. (2023). Trastuzumab deruxtecan in metastatic breast cancer with variable HER2 expression: the phase 2 DAISY trial. Nat. Med. *29*, 2110–2120.
51. Robbins, C.J., Fernandez, A.I., Han, G., Wong, S., Harigopal, M., Podoll, M., Singh, K., Ly, A., Kuba, M.G., Wen, H., et al. (2023). Multi-institutional Assessment of Pathologist Scoring HER2 Immunohistochemistry. Mod. Pathol. *36*, 100032.
52. Zeileis, A., and Hothorn, T. (2002). Diagnostic checking in regression relationships. R. News *2*, 7–10.
53. Gesmann, M., and de Castillo, D. (2011). googleVis: interface between R and the Google visualisation API. R. J. *3*, 40–44.
54. Therneau, T.M., and Grambsch, P.M. (2000). The Cox Model. In Modeling Survival Data: Extending the Cox Model Statistics for Biology and Health (Springer), pp. 39–77.
55. Symmans, W.F., Wei, C., Gould, R., Yu, X., Zhang, Y., Liu, M., Walls, A., Bousamra, A., Ramineni, M., Sinn, B., et al. (2017). Long-Term Prognostic Risk After Neoadjuvant Chemotherapy Associated With Residual Cancer Burden and Breast Cancer Subtype. J. Clin. Orthod. *35*, 1049–1060.
56. Cardoso, F., van't Veer, L.J., Bogaerts, J., Slaets, L., Viale, G., Delaloge, S., Pierga, J.-Y., Brain, E., Causeret, S., DeLorenzi, M., et al. (2016). 70-Gene Signature as an Aid to Treatment Decisions in Early-Stage Breast Cancer. N. Engl. J. Med. *375*, 717–729.
57. Piccart, M., van 't Veer, L.J., Poncet, C., Lopes Cardozo, J.M.N., Delaloge, S., Pierga, J.-Y., Vuylsteke, P., Brain, E., Vrijaldenhoven, S., Neijenhuis, P.A., et al. (2021). 70-gene signature as an aid for treatment decisions in early breast cancer: updated results of the phase 3 randomised MINDACT trial with an exploratory analysis by age. Lancet Oncol. *22*, 476–488.
58. Berry, D.A. (2011). Adaptive clinical trials in oncology. Nat. Rev. Clin. Oncol. *9*, 199–207.
59. Park, J.W., Liu, M.C., Yee, D., Yau, C., van 't Veer, L.J., Symmans, W.F., Paoloni, M., Perlmutter, J., Hylton, N.M., Hogarth, M., et al. (2016). Adaptive Randomization of Neratinib in Early Breast Cancer. N. Engl. J. Med. *375*, 11–22.
60. Rugo, H.S., Olopade, O.I., DeMichele, A., Yau, C., van 't Veer, L.J., Buxton, M.B., Hogarth, M., Hylton, N.M., Paoloni, M., Perlmutter, J., et al. (2016). Adaptive Randomization of Veliparib–Carboplatin Treatment in Breast Cancer. N. Engl. J. Med. *375*, 23–34.
61. Oken, M.M., Creech, R.H., Tormey, D.C., Horton, J., Davis, T.E., McFadden, E.T., and Carbone, P.P. (1982). Toxicity and response criteria of the Eastern Cooperative Oncology Group. Am. J. Clin. Oncol. *5*, 649–655.
62. Chien, A.J., Tripathy, D., Albain, K.S., Symmans, W.F., Rugo, H.S., Melisko, M.E., Wallace, A.M., Schwab, R., Helsten, T., Forero-Torres, A.,

CellPress
OPEN ACCESS

et al. (2020). MK-2206 and Standard Neoadjuvant Chemotherapy Improves Response in Patients With Human Epidermal Growth Factor Receptor 2–Positive and/or Hormone Receptor–Negative Breast Cancers in the I-SPY 2 Trial. J. Clin. Orthod. *38*, 1059–1069.

63. Nanda, R., Liu, M.C., Yau, C., Shatsky, R., Pusztai, L., Wallace, A., Chien, A.J., Forero-Torres, A., Ellis, E., Han, H., et al. (2020). Effect of Pembrolizumab Plus Neoadjuvant Chemotherapy on Pathologic Complete Response in Women With Early-Stage Breast Cancer: An Analysis of the Ongoing Phase 2 Adaptively Randomized I-SPY 2 Trial. JAMA Oncol. *6*, 676–684.

64. Pusztai, L., Yau, C., Wolf, D.M., Han, H.S., Du, L., Wallace, A.M., String-Reasor, E., Boughey, J.C., Chien, A.J., Elias, A.D., et al. (2021). Durvalumab with olaparib and paclitaxel for high-risk HER2-negative stage II/III breast cancer: Results from the adaptively randomized I-SPY 2 trial. Cancer Cell *39*, 989–998.e5.

65. Espina, V., Wulfkuhle, J.D., Calvert, V.S., VanMeter, A., Zhou, W., Coukos, G., Geho, D.H., Petricoin, E.F., and Liotta, L.A. (2006). Laser-capture microdissection. Nat. Protoc. *1*, 586–603.

66. Sheehan, K.M., Calvert, V.S., Kay, E.W., Lu, Y., Fishman, D., Espina, V., Aquino, J., Speer, R., Araujo, R., Mills, G.B., et al. (2005). Use of Reverse Phase Protein Microarrays and Reference Standard Development for Molecular Network Analysis of Metastatic Ovarian Carcinoma. Mol. Cell. Proteomics *4*, 346–355.

67. Akbani, R., Becker, K.-F., Carragher, N., Goldstein, T., de Koning, L., Korf, U., Liotta, L., Mills, G.B., Nishizuka, S.S., Pawlak, M., et al. (2014). Realizing the Promise of Reverse Phase Protein Arrays for Clinical, Translational, and Basic Research: A Workshop Report. Mol. Cell. Proteomics *13*, 1625–1643.

68. Signore, M., and Reeder, K.A. (2012). Antibody Validation by Western Blotting. In Molecular Profiling Methods in Molecular Biology, V. Espina and L.A. Liotta, eds. (Humana Press), pp. 139–155.

69. Gallagher, R.I., Silvestri, A., Petricoin, E.F., Liotta, L.A., and Espina, V. (2011). Reverse Phase Protein Microarrays: Fluorometric and Colorimetric Detection. In Protein Microarray for Disease Analysis, C.J. Wu, ed. (Humana Press), pp. 275–301.

70. Pin, E., Federici, G., and Petricoin, E.F. (2014). Preparation and Use of Reverse Protein Microarrays. Curr. Protoc. Protein Sci. *75*, 27.7.1–27.7.29.

71. Huang, D.W., Sherman, B.T., and Lempicki, R.A. (2009). Systematic and integrative analysis of large gene lists using DAVID bioinformatics resources. Nat. Protoc. *4*, 44–57.

STAR★METHODS

KEY RESOURCES TABLE

REAGENT or RESOURCE	SOURCE	IDENTIFIER
Biological samples		
Tumor biopsy before treatment	I-SPY 2 TRIAL	https://clinicaltrials.gov/ct2/show/NCT01042379
Critical commercial assays		
Reverse phase protein array (RPPA)	Petricoin Lab, George Mason University	https://www.ncbi.nlm.nih.gov/geo/query/acc.cgi?acc=GPL28470
Deposited data		
Raw and processed RPPA data	This study	*Gene Expression Omnibus (GEO)* SubSeries GSE196093 (RPPA) (https://www.ncbi.nlm.nih.gov/geo/query/acc.cgi?acc=GSE196093, as part of the SuperSeries GSE196096 (https://www.ncbi.nlm.nih.gov/geo/query/acc.cgi?acc=GSE196096); and in the I-SPY 2 Google Cloud repository (http://www.ispytrials.org/results/data)
Patient-level expression signature and clinical data	This study	*Gene Expression Omnibus (GEO)* SuperSeries GSE196096 (https://www.ncbi.nlm.nih.gov/geo/query/acc.cgi?acc=GSE196096); and in the I-SPY 2 Google Cloud repository (http://www.ispytrials.org/results/data)
Software and algorithms		
stats R package (v.3.6.3)	R Core Team (2020)	https://stat.ethz.ch/R-manual/R-devel/library/stats/html/stats-package.html
lmtest R package (v.0.937)	Zeileis et al., 2002[52]	https://CRAN.R-project.org/package=lmtest
googleVis R package (v.0.6.4)	Gesmann and de Castillo, 2011[53]	https://CRAN.R-project.org/package=googleVis
survival R package (v.3.1–12)	Therneau et al., 2000[54]	https://CRAN.R-project.org/package=survival
forestplot R package (version 2.0.1)	Max Gordon	https://CRAN.R-project.org/package=forestplot

RESOURCE AVAILABILITY

Lead contact

Further information and requests for resources or data should be directed to and will be fulfilled by Rosa Isela Gallagher (rgallag3@gmu.edu)

Materials availability

This study did not generate new unique reagents.

Data and code availability

- Protein/phosphoprotein and clinical data used in this study is available in NCBI's *Gene Expression Omnibus* (GEO) SuperSeries GSE196096 (https://www.ncbi.nlm.nih.gov/geo/query/acc.cgi?acc=GSE196096) and its two SubSeries and GSE196093 (RPPA: https://www.ncbi.nlm.nih.gov/geo/query/acc.cgi?acc=GSE196093), and through the I-SPY 2 Google Cloud repository (www.ispytrials.org/results/data). Data on GEO represents the data as currently recorded in our database.
- Additional de-identified subject level data may be requested by qualified investigators. Details of the trial, data, contact information, proposal forms, and review and approval process are available at the following website: https://www.ispytrials.org/collaborate/proposal-submissions.
- This paper does not report original code.
- Any additional information required to reanalyze the data reported in this work paper is available from the Lead Contact upon request.

EXPERIMENTAL MODEL AND STUDY PARTICIPANT DETAILS

I-SPY 2 TRIAL overview

I-SPY 2 is an ongoing, open-label, adaptive, randomized phase II, multicenter trial of neoadjuvant therapy for early-stage breast cancer (NCT01042379; IND 105139). This platform trial evaluates multiple investigational arms in parallel against a common standard of care control arm. The primary endpoint is pCR (ypT0/is, ypN0), defined as the absence of invasive cancer in the breast and regional nodes at the time of surgery.[55] As I-SPY 2 is modified intent-to-treat, patients receiving any dose of study therapy are considered evaluable; those who switch to non-protocol therapy, progress, forgo surgery, or withdraw are deemed 'non-pCR'. Secondary analytes include residual cancer burden (RCB) and event-free and distant relapse-free survival (EFS and DRFS).[55]

Trial design

Assessments at screening establish eligibility and classify participants into subtypes defined by hormone receptor (HR) status, HER2, and 70-gene signature (Mammaprint) status.[56,57] Adaptive randomization in I-SPY 2 preferentially assigns patients to trial arms according to continuously updated Bayesian probabilities of pCR rates within each biomarker signature; 20% of patients are randomly assigned to the control arm.[58] While accrual is ongoing, a statistical engine assesses the accumulating pathologic and MRI responses at weeks 3 and 12 and continuously re-estimates the probabilities of an experimental arm being superior to the control in each defined biomarker signature. An arm can be dropped for futility if the predicted probability of success in a future 300-patient, 1:1 randomized, phase 3 trial drops below 10%, or graduate for efficacy if the probability of success reaches 85% or greater in any biomarker signature. The clinical control arm for the efficacy analysis uses patients randomized throughout the entire trial. Experimental arms have variable sample sizes: highly effective therapies graduate with fewer patients in the experimental arm; arms that are equal to, or marginally better than, the control arm accrue slower and are stopped if they have not graduated, or terminated for lack of efficacy, before reaching a sample size of 75. During the design of each new experimental arm the investigators together with the pharmaceutical sponsor decide in which of the 10 *a priori* defined biomarker signatures the drug will be tested. Upon entry to the trial, participants are dichotomized into hormone receptor (HR) negative versus positive, HER2 positive versus negative, and MammaPrint High1 [MP1] versus High2 [MP2] status. From these 8 biomarker combinations ($2 \times 2 \times 2$) I-SPY has created 10 biomarker signatures that represent the disease subsets of interest (e.g., all patients, all HR+, all HER2+, HR+/HER2-, etc, for complete list see ref. [58]) in which a drug can be tested for efficacy. Efficacy is monitored in each of these biomarker signatures separately and an arm could graduate in any or all biomarker signature of interest. When graduation occurs, accrual to the arm stops, final efficacy results are updated when all pathology results are complete. The final estimated pCR results therefore may differ from the predicted pCR rate at the time of graduation. Additional details on the study design have been published elsewhere.[59,60]

Eligibility

Participants eligible for I-SPY 2 are women >18 years of age with stage II or III breast cancer with a minimum tumor size of >2 · 5 cm by clinical exam, or >2 · 0 cm by imaging, and Eastern Cooperative Oncology Group performance status of 0 or 1.[61] HR-positive/HER2-negative cancers assessed as low risk by the 70-gene MammaPrint test are ineligible as they receive little benefit from systemic chemotherapy.

Treatment

This correlative study involved 736 women with high-risk stage II and III early breast cancer who were enrolled in the first 8 experimental arms of I-SPY 2 plus concurrent controls as shown in the schema of Figure 1A. All patients received at least standard chemotherapy (paclitaxel alone followed by doxorubicin/cyclophosphamide (T- > AC; or with trastuzumab (H) in HER2+, T + H- > AC)) or in combination (taxane phase) with investigational agents: veliparib/carboplatin (VC; HER2-only: VC - > AC); neratinib (N; All patients: T + N- > AC); MK2206 (M; HER2-: T + M- > AC; HER2+: T + H + M- > AC); Ganitumab (GM: HER2-only: T + GM- > AC); AMG386 (HER2-: T + AMG386->AC; HER2+: T + H + AMG386->AC); TDM1/pertuzumab (P) (HER2+: TDM1/P- > AC); HP (HER2+: T + HP- > AC); and a PD1 inhibitor (PD1-inh; HER2-: T + PD1-inh->AC). For HER2+ patients, N was administered instead of H, whereas M and AMG386 were administered in addition to H. Dose reductions and toxicity management were specified in the protocol. Adverse events were collected according to the NCI Common Terminology Criteria for Adverse Events (CTCAE) version 4.0. After completion of AC, patients underwent lumpectomy or mastectomy and nodal sampling, with choice of surgery at the discretion of the treating surgeon. Detailed descriptions of the design, eligibility, and efficacy of these 8 experimental arms of the I-SPY 2 trial have been reported previously.[10,59,60,62–64]

Trial Oversight

I-SPY 2 is conducted in accordance with the guidelines for Good Clinical Practice and the Declaration of Helsinki, with approval for the study protocol and associated amendments obtained from independent ethics committees at each site. Written, informed consent was obtained from each participant prior to screening and again prior to treatment. The I-SPY 2 Data Safety Monitoring Board meets monthly to review patient safety.

METHOD DETAILS

Pretreatment biopsy microdissection and molecular profiling

Core needle biopsies of 16-gauge were taken from the patient's primary breast tumor before treatment. Collected tissue samples were immediately frozen in Tissue-Tek O.C.T. embedding media and then stored at - 80°C until further processing. Enriched epithelial cell populations were isolated from 8 μM cryosections of tissue using an Arcturus Pixcell IIe LCM system (Arcturus, Mountain View, CA, USA).[65] Approximately 10,000 epithelial cells were captured for each sample at the pre-treatment time point. Microdissected material was stored at −80°C and samples were lysed in extraction buffer composed of Tissue Protein Extraction Reagent (TPER; ThermoFisher), 2x SDS-PAGE Sample Buffer (ThermoFisher) mixed 1:1 and 2.5% beta-mercaptoethanol (BME) per 1 mL at a concentration of approximately 500 cells per 1μL of extraction buffer. Samples were heated at 100°C for 5min, brought to room temperature, briefly centrifuged and then stored at −20°C until ready for printing. Cell lysates were printed in triplicate spots (approx. 10nL per spot) onto nitrocellulose coated slides (Grace Biolabs, Bend, OR, USA) using a Quanterix 2470 Arrayer (Quanterix, Billerica, MA, USA). Standard curves of control cell lysates were included for quality assurance purposes.[66] The proteins and phosphoproteins measured in this study (analytes, 139 in total) are listed in Table S5. Antibodies used on the arrays were validated before use by confirming the presence of a single band at the appropriate molecular weight with a panel of control cell lysates using conventional western blotting.[67,68] Immunostaining was performed by probing each slide with one primary antibody targeting the protein of interest.[69,70] Biotinylated goat anti-rabbit IgG (H + L) (1:7,500, Vector Laboratories Inc, Burlingame, CA) or rabbit anti-mouse IgG (1:10, DakoCytomation, Carpinteria, CA, USA) were used as secondary antibodies. Signal amplification was performed using a tyramide-based avidin/biotin amplification system (DakoCytomation, Carpinteria, CA, USA) followed by streptavidin-conjugated IRDye 680 (LI-COR, Lincoln, NE, USA) for visualization. Negative controls were stained with secondary antibody alone. Total protein was measured using Sypro Ruby protein blot staining per manufacturer's instructions (Molecular Probes, Eugene, OR, USA).

RPPA data analysis

RPPA data was generated directly from images acquired using a Tecan PowerScanner (Tecan, Mannedorf, Switzerland) and analyzed with MicroVigene software Version 5.1.0.0 (Vigenetech, Carlisle, MA, USA).[70] Total protein intensities for each sample were calculated by averaging the Sypro staining intensity of the three replicate spots. For each sample/endpoint the final signal intensity was calculated by: 1) subtraction of negative control spot intensity from primary antibody spot intensity, 2) averaging the resulting net intensities for the three replicate spots, and 3) dividing by the total protein intensity value for each sample. To remove batch effects we standardized each array prior to combining, by (1) sampling 5000 times, maintaining a receptor subtype balance equal to that of the first ~1000 patients (HR + HER2-: 0.384, TN:0.368, HR + HER2+:0.158, HR-HER2+:0.09); (2) calculating the mean(mean) and mean(sd) for each RPPA endpoint; (3) z-scoring each endpoint using the calculated mean/sd from (2), as described previously.[14] Normalized and raw RPPA data over all analytes for the 736 patients with RPPA analysis in this study are part of of the I-SPY2-990 mRNA/RPPA data resource deposited in NCBI's *Gene Expression Omnibus* (GEO) and on the I-SPY 2 Google Cloud repository (https://console.cloud.google.com/storage/browser/wolf_et_al_2021_ispy2_subtypes_990a).[6]

QUANTIFICATION AND STATISTICAL ANALYSIS

Statistical analysis of continuous RPPA biomarkers

Unsupervised clustering was performed using Pearson correlation and complete linkage. We assessed association between each continuous biomarker and response in the population as a whole and within each arm and HR/HER2 subtype using a logistic model. In whole-population analyses, models were adjusted for HR, HER2, and treatment arm (pCR~ biomarker + HR + HER2 + Tx). Within treatment arms, models were adjusted for HR and HER2 as appropriate. Markers are analyzed individually; likelihood ratio (LR) p values are descriptive. We employed Benjamini-Hochberg (BH) multiple testing correction,[71] with a significance threshold of BH $p < 0.05$ to all experimental results, and reported p values as BH or uncorrected as appropriate. Analyses and visualizations were performed in the computing environment R (v.3.6.3) using R Packages 'stats' (v.3.6.3), and 'lmtest' (v.0.9–37).

RPPA cluster definition

To define the RPPA clusters we performed unsupervised clustering on the continuous RPPA data using Pearson correlation and complete linkage, using the threshold 1.54 to partition the dendrogram into 10 clusters (function hclust2treeview from R package ctc and functions as.dendrogram and cutree from the base R package stats). Cluster 7 (132 patients) was then further partitioned into two sub-clusters, 7a (16 patients) and 7b, based on the observation that 7a forms a HER2-signaling-enriched coherent subgroup. To visualize the protein/phosphoprotein profiles characterizing each RPPA cluster, we calculated the mean value of each endpoint in each cluster and displayed the results in a heatmap. Sankey plots showing relationships between RPPA clusters and other categorical variables were generated using googleVis (v.0.6.4).

Survival analyses

Cox proportional hazards modeling was used to estimate DRFS hazard ratios for pCR within each RPPA cluster, visualized differences in survival between responders and non-responders were visualized using Kaplan-Meier plots. We also used Cox proportional hazards modeling to assess association between the levels of individual RPPA protein/phosphoprotein analytes and DRFS within non-responders in each RPPA cluster in a model adjusting for HR and HER2 status. Resulting p values were adjusted for multiple

hypothesis testing using the Benjamini-Hochberg method. These analyses were performed using the coxph and Surv functions within the R package survival.[54] The hazard ratio forest plot was generated using the R package forestplot (version 2.0.1).

HARPS signature determination

Optimal cut points of biomarker positivity for EGFR Y1173 and ERBB2 Y1248 in the TN patient population treated with neratinib were determined by receiver-operating characteristic analysis (ROC) using Youden Index methodology.[14] These cut point values were extrapolated to the full normalized TN dataset to assess HARPS positivity in the TN population across all arms of the I-SPY 2 TRIAL.

ADDITIONAL RESOURCES

More information about the I-SPY 2 platform trial (NCT01042379) and associated resources can be found at https://clinicaltrials.gov/ct2/show/NCT01042379, https://www.ispytrials.org/i-spy-platform/i-spy2 and https://ispypatient.org. This study is registered with ClinicalTrials.gov: NCT01042379.

Cell Reports Medicine, Volume *4*

Supplemental information

Protein signaling and drug target activation signatures to guide therapy prioritization: Therapeutic resistance and sensitivity in the I-SPY 2 Trial

Rosa I. Gallagher, Julia Wulfkuhle, Denise M. Wolf, Lamorna Brown-Swigart, Christina Yau, Nicholas O'Grady, Amrita Basu, Ruixiao Lu, Michael J. Campbell, Mark J. Magbanua, Jean-Philippe Coppé, I-SPY 2 Investigators, Smita M. Asare, Laura Sit, Jeffrey B. Matthews, Jane Perlmutter, Nola Hylton, Minetta C. Liu, W. Fraser Symmans, Hope S. Rugo, Claudine Isaacs, Angela M. DeMichele, Douglas Yee, Paula R. Pohlmann, Gillian L. Hirst, Laura J. Esserman, Laura J. van `t Veer, and Emanuel F. Petricoin

Protein signaling and drug target activation signatures to guide therapy prioritization: therapeutic resistance and sensitivity in the I-SPY 2 TRIAL

Rosa I. Gallagher, Julia Wulfkuhle, Denise M. Wolf, Lamorna Brown-Swigart, Christina Yau, Nicholas O'Grady, Amrita Basu, Ruixiao Lu, Michael J. Campbell, Mark J. Magbanua, Jean-Philippe Coppé, I-SPY2 Investigators, Smita M. Asare, Laura Sit, Jeffrey B. Matthews, Jane Perlmutter, Nola Hylton, Minetta C. Liu, W. Fraser Symmans, Hope S. Rugo, Claudine Isaacs, Angela M. DeMichele, Douglas Yee, Paula R. Pohlmann, Gillian L. Hirst, Laura J. Esserman, Laura J. van 't Veer, Emanuel F. Petricoin

SUPPLEMENTAL INFORMATION

Additional resources

More information about the I-SPY 2 platform trial (NCT01042379) and associated resources can be found at https://clinicaltrials.gov/ct2/show/NCT01042379, https://www.ispytrials.org/i-spy-platform/i-spy2 and https://ispypatient.org.

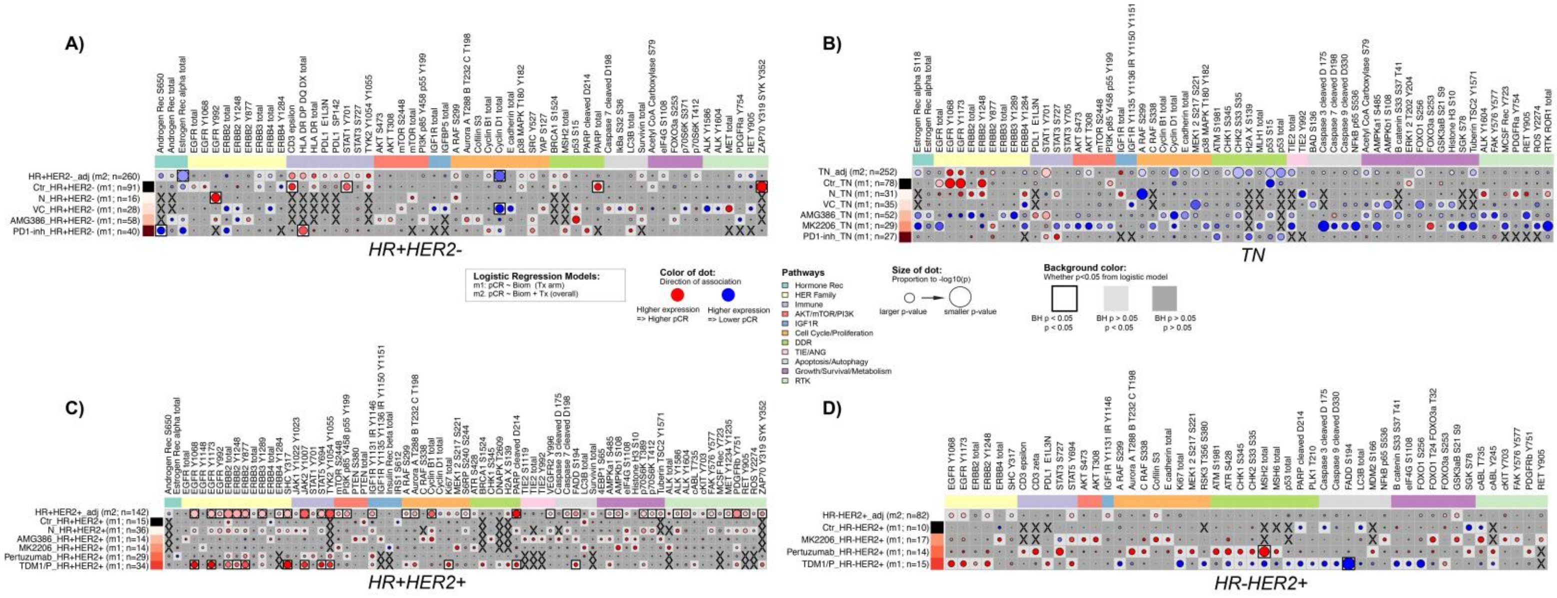

Figure S1. Dot plots of Protein/phosphoprotein expression/activation levels associated with HR/HER2 subtype and pCR. related to Figure 3. Dot plots of A) HR+HER2- subtype, B) Triple negative (TN) subtype, C) HR+HER2+ subtype and D) HR-HER2+ subtype.

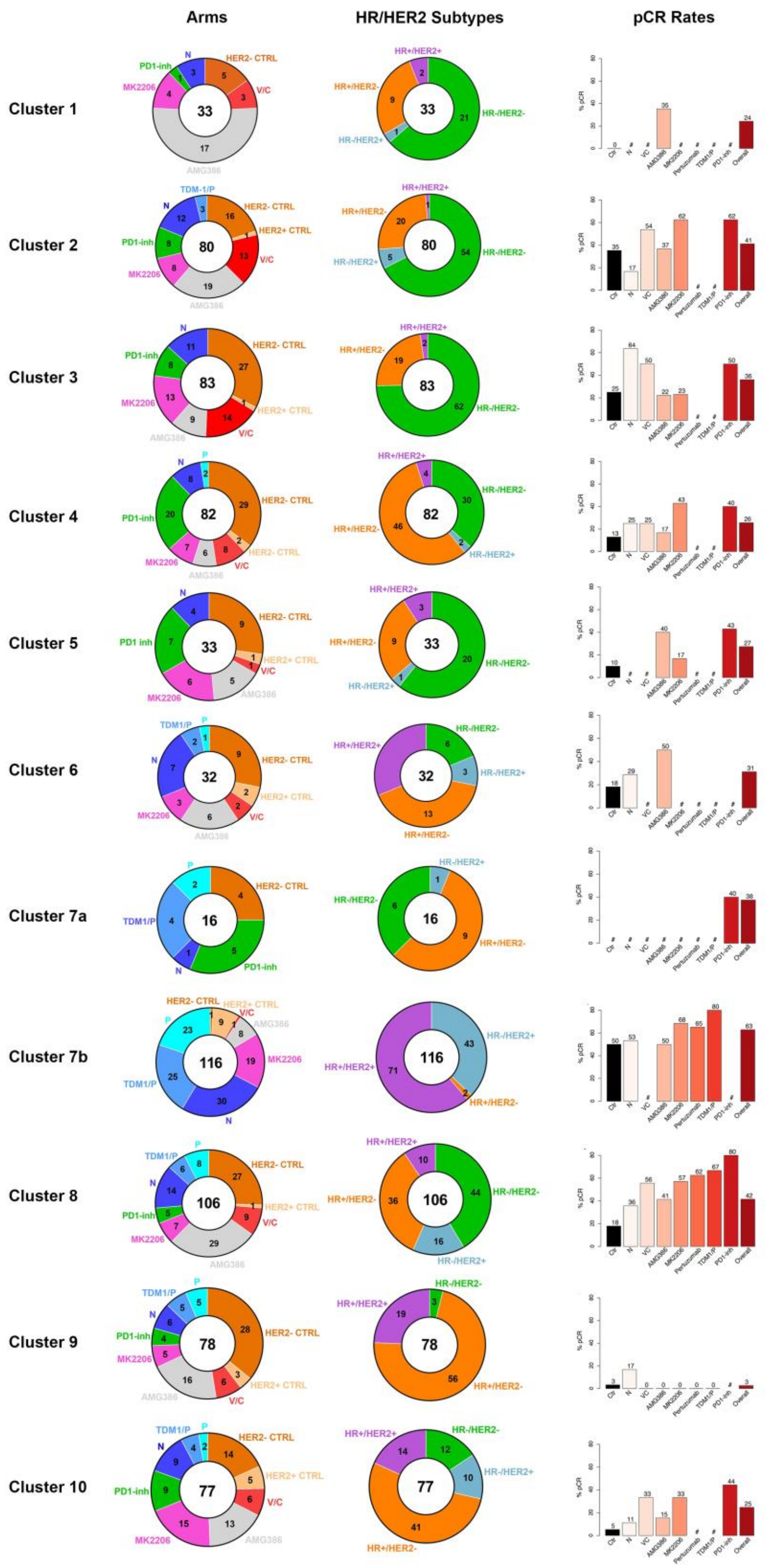

Figure S2. Treatment arm, HR/HER2 subgroup distribution and pCR rates for the 11 protein/phosphoprotein-driven cluster signatures identified, related to Figure 4. Donut plots of treatment arm composition (left), HR/HER2 subtype composition (middle) and pCR rates for each of the therapeutic arms (right) are shown.

	HR+HER2-	TN	HR+HER2+	HR-HER2+	Total
Ctr	91	78	15	10	**194**
N	16	31	36	22	**105**
VC	28	35	0	0	**63**
AMG386	58	52	14	4	**128**
MK2206	27	29	14	17	**87**
Pertuzumab	0	0	29	14	**43**
TDM1/P	0	0	34	15	**49**
PD1-inh	40	27	0	0	**67**
Total	**260**	**252**	**142**	**82**	**736**

Table S1. Patient distribution between arms and HR/HER2 subtypes in the RPPA dataset, related to Figure 1.
